# High-dose influenza vaccine augments serological and cellular immunity of older people with HIV

**DOI:** 10.1172/jci.insight.199232

**Published:** 2026-03-05

**Authors:** Jonah Kupritz, Sheldon Davis, TianHao Liu, Prabhsimran Singh, Daniel Andrés Díaz-Pachón, Allan Rodriguez, Scott D. Boyd, Rajendra Pahwa, Suresh Pallikkuth, Savita G. Pahwa

**Affiliations:** 1 Department of Microbiology and Immunology,; 2 Division of Biostatistics, Department of Public Health Sciences; and; 3 Division of Infectious Diseases, Department of Medicine, University of Miami Miller School of Medicine, Miami, Florida, USA.; 4 Department of Pathology, Stanford University School of Medicine, Stanford, California, USA.

**Keywords:** AIDS/HIV, Immunology, Infectious disease, Clinical trials, Influenza, Vaccines

## Abstract

**BACKGROUND:**

High-dose influenza vaccine, containing 4 times more antigen than the standard dose, is recommended for people 65 years or older, but there is a knowledge gap surrounding its effect in people with HIV (PWH), who remain more vulnerable to serious influenza infections than people without HIV (PWoH) despite virological suppression. Our primary goal was to assess whether the high-dose vaccine improves antibody responses in PWH, especially older PWH.

**METHODS:**

We assessed antibody responses to sequential high- versus standard-dose influenza vaccination in PWH. Young (18–40 years) PWoH (*n* = 55) and PWH (*n* = 37) and older (≥60 years) PWoH (*n* = 72) and PWH (*n* = 67) received a standard-dose vaccine during the 2020–2024 seasons, and 123 participants (41 older PWH) received a high-dose vaccine the subsequent season. All PWH were virologically suppressed on antiretroviral therapy. HA inhibition (HAI) titer and HA-specific IgG were analyzed at 0 to 180 days after vaccination; T cell activation–induced responses were assessed by flow cytometry.

**RESULTS:**

All groups mounted significant HAI and IgG responses to all vaccine antigens at 28 days after standard- and high-dose vaccination. Responses to A/H1N1 were lower in magnitude and durability in older PWH compared with young PWoH after the standard dose and were not boosted with the high dose, whereas the high dose enhanced A/H3N2 and B/Victoria IgG, in addition to CD4^+^ T cell responses to all antigens, in older PWH.

**CONCLUSION:**

Our data demonstrate partial efficacy of a high-dose vaccine in augmenting antibody responses of older PWH while highlighting limitations in boosting A/H1N1-specific responses.

**TRIAL REGISTRATION:**

ClinicalTrials.gov NCT04487041.

**FUNDING:**

NIH grant (5R01AG068110).

## Abstract 2

High-dose vaccination strengthened immunity in older adults with HIV after prior standard dosing, but not all strain-specific weaknesses were overcome.

## Introduction

Aging is accompanied by a reduction in the magnitude and quality of protective immune responses to infection or vaccination (1). Age-associated immune decline, or immunosenescence, is characterized by low-grade inflammation and the accumulation of terminally differentiated or senescent-like immune cells (2, 3). Various stressors, including low-level viral replication and long-term antiretroviral therapy (ART), may induce premature immunosenescence and accelerate the onset of aging-associated comorbidities in people with HIV (PWH) (4, 5). There are roughly 1.2 million PWH in the United States, more than half of whom are over the age of 50 years, and the global population of PWH 50 years or older is estimated to exceed 8 million (6).

Influenza is a respiratory illness caused by influenza viruses, which affects up to 10% of the global population and causes more than 250,000 deaths annually (7). It is estimated that more than half of influenza-related hospitalizations and up to 85% of influenza-related deaths occur in people 65 years or older (8, 9). Seasonal influenza outbreaks are caused by viruses of the influenza A (A/H1N1 and A/H3N2) and B (B/Victoria and B/Yamagata) lineages; circulation of B/Yamagata is thought to have ceased during the COVID-19 pandemic (10). In a large study of people hospitalized with influenza infection, A/H1N1 and B strain infections were associated with more severe outcomes, including intensive care unit admission and death, compared with A/H3N2 infections (11).

Among people of all ages, vaccination is the mainstay of influenza prevention. Influenza vaccines are available in live attenuated, inactivated (egg-based or mammalian cell–based), or recombinant formats. Vaccine effectiveness wanes appreciably with age. In recent influenza seasons for which detailed data are available, effectiveness against any influenza infection was found to decline from approximately 50% among children younger than 18 years to around 30% among adults aged 65 years or older (12, 13). To address the lower vaccine effectiveness and increased rates of serious influenza outcomes, high-dose inactivated influenza vaccine, containing 4 times the amount of antigen as the standard-dose vaccine, is recommended for all people aged 65 years or older (14). Compared with the standard-dose vaccine, the high-dose vaccine is reported to reduce influenza infection and hospitalization by 15%–25%, although effectiveness varies by viral strain and antigenic match between the vaccine and circulating viruses (15, 16). Adjuvanted formulations, which comprise less than 10% of influenza vaccines administered annually in the United States, may further enhance protection relative to unadjuvanted high-dose vaccine (17). We have previously reported that antibody responses to inactivated, nonadjuvanted seasonal standard-dose influenza vaccine are impaired at an earlier age among virally suppressed PWH and to a greater extent than in age-matched people without HIV (PWoH) (18–20). Clinically, PWH are at a higher risk of developing severe complications from influenza, although the risk is greatly attenuated with suppressive ART (21, 22). However, annual vaccination guidelines do not currently address the specific situation of virally suppressed PWH. Vaccine responses may be biased toward influenza strains or subtypes depending on an individual’s preexisting immune landscape, underscoring the importance of studying influenza vaccine responses in demographically diverse populations (23, 24). Whether high-dose vaccination can overcome preexisting subtype immunodominance or cell-intrinsic immune deficits that persist in well-suppressed HIV infection remains to be determined.

There is a gap in knowledge about standard- and high-dose vaccine effectiveness in PWH and particularly in older PWH, a rapidly growing population due to the increased life expectancy afforded by effective ART. A 2-arm randomized controlled trial of younger and middle-aged PWH (median age of high-dose recipients: 44 years) found that a high-dose vaccine slightly improved rates of seroprotection against A/H1N1 and B/Victoria during the 2010–2011 influenza season (24), and a retrospective cohort study reported reduced rates of influenza-like illness among PWH (mean age of high-dose recipients: 49.9 years) who received a high-dose vaccine during the 2017–2018 influenza season (25). To our knowledge, no study to date has comparatively evaluated immune responses to sequential standard- and high-dose seasonal influenza vaccination among PWH aged 60 years and older, a population that faces the independent and cumulative immunosuppressive effects of aging and chronic HIV infection. To address these knowledge gaps and to inform better protection of PWH, we conducted a crossover clinical trial of standard- and high-dose influenza vaccine responses in young and older PWoH and virally suppressed PWH, including PWH aged 60 years or older, employing 3 independent readouts of immunogenicity: HA inhibition (HAI) titer, quantitative HA-specific IgG, and CD4^+^/CD8^+^ T cell activation–induced marker responses. Measuring both HAI and HA-specific IgG provided complementary insights into vaccine-induced immunity by capturing both the functional neutralizing capacity of antibodies (HAI) and the total antigen-specific antibody response (IgG), enabling a more complete assessment of immunogenicity and circumventing HAI assay limitations, including interlaboratory variability and lack of hemagglutination with contemporary A/H3N2 strains (26, 27). In our study, a total of 231 participants were administered a standard-dose vaccine; the following influenza season, 123 participants (53.2%), including 41 older PWH, returned to receive a high-dose vaccine. Serological responses were measured at the prevaccination time point and at the postvaccination (dpv) time points of 7, 14, 28, and 180 days, and T cell responses were assessed at 0 and 14 dpv, to elucidate the influence of aging and of suppressed HIV infection on the magnitude and kinetics of strain-specific serological and cellular responses to influenza vaccination and to evaluate the relative benefit of a high-dose vaccine in the population of older PWH.

## Results

### Standard-dose influenza vaccination drives a robust serum response to influenza antigens, which wanes from 28 to 180 dpv.

**Figure 1:**
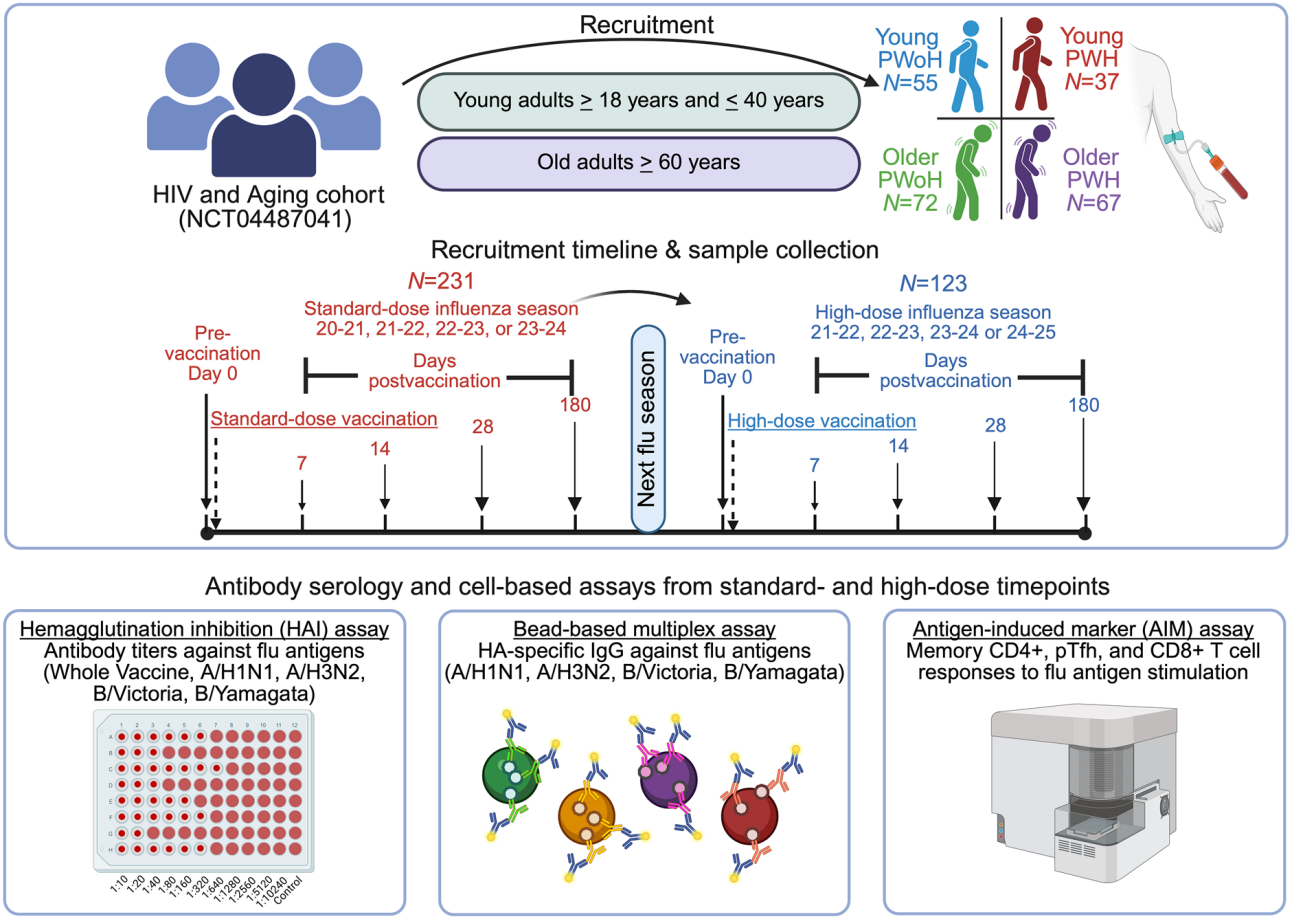
Graphical overview of the influenza vaccine study design.

In total, 231 participants were enrolled in the study and received a standard-dose influenza vaccine. Young (18–40 years) PWoH (*n* = 55) and PWH (*n* = 37) along with older (≥60 years) PWoH (*n* = 72) and PWH (*n* = 67) were administered a standard-dose vaccine during one of the 2020–2024 influenza seasons (Figure 1 and Table 1). Median ages were similar across HIV status within each age group: 30 versus 34 years for young PWoH and PWH, respectively, and 63 versus 64 years for older PWoH and PWH, respectively (Supplemental Figure 1; supplemental material available online with this article; https://doi.org/10.1172/jci.insight.199232DS1). Influenza vaccination at any time point prior to study enrollment was reported by more than 60% of participants in each group (young PWoH, 64%; young PWH, 70%; older PWoH, 68%; and older PWH, 88%; *P* = 0.27). Female participants comprised the majority (58%) of the young PWoH group, and 27%–30% of the other 3 groups (*P* = 0.002). Median time since HIV-1 diagnosis was 7.3 and 20.7 years and median duration of ART was 6.5 and 20.0 years for young and older PWH, respectively. All PWH had an undetectable HIV-1 viral load for at least 1 year prior to study enrollment. All groups, including young and older PWH, had a mean absolute CD4^+^ T cell count greater than 500 and mean CD4/CD8 ratio greater than 1. Self-reported cardiometabolic comorbidity data were available for most participants (young PWoH, *n* = 52; young PWH, *n* = 28; older PWoH, *n* = 52; older PWH, *n* = 52), revealing an expected age-associated increase in hypertension, diabetes, hyperlipidemia, and cardiovascular disease prevalence and no clear differences between participants with and without HIV (Supplemental Table 1).

At baseline, we observed higher frequencies of seroprotective titers (≥1:40) across all groups against B/Yamagata (89%–99%) and A/H1N1 (90%–94%) strains, compared with B/Victoria (60%–79%), with no significant intergroup differences. Titers against A/H3N2 were available only for a subset of participants (*n* = 115) vaccinated during the 2020–2021 and 2021–2022 influenza seasons due to technical limitations of the A/H3N2 HAI assay during recent seasons (27); among these individuals, 75%–94% had seroprotective A/H3N2 titers at baseline (Supplemental Table 2).

**Figure 2:**
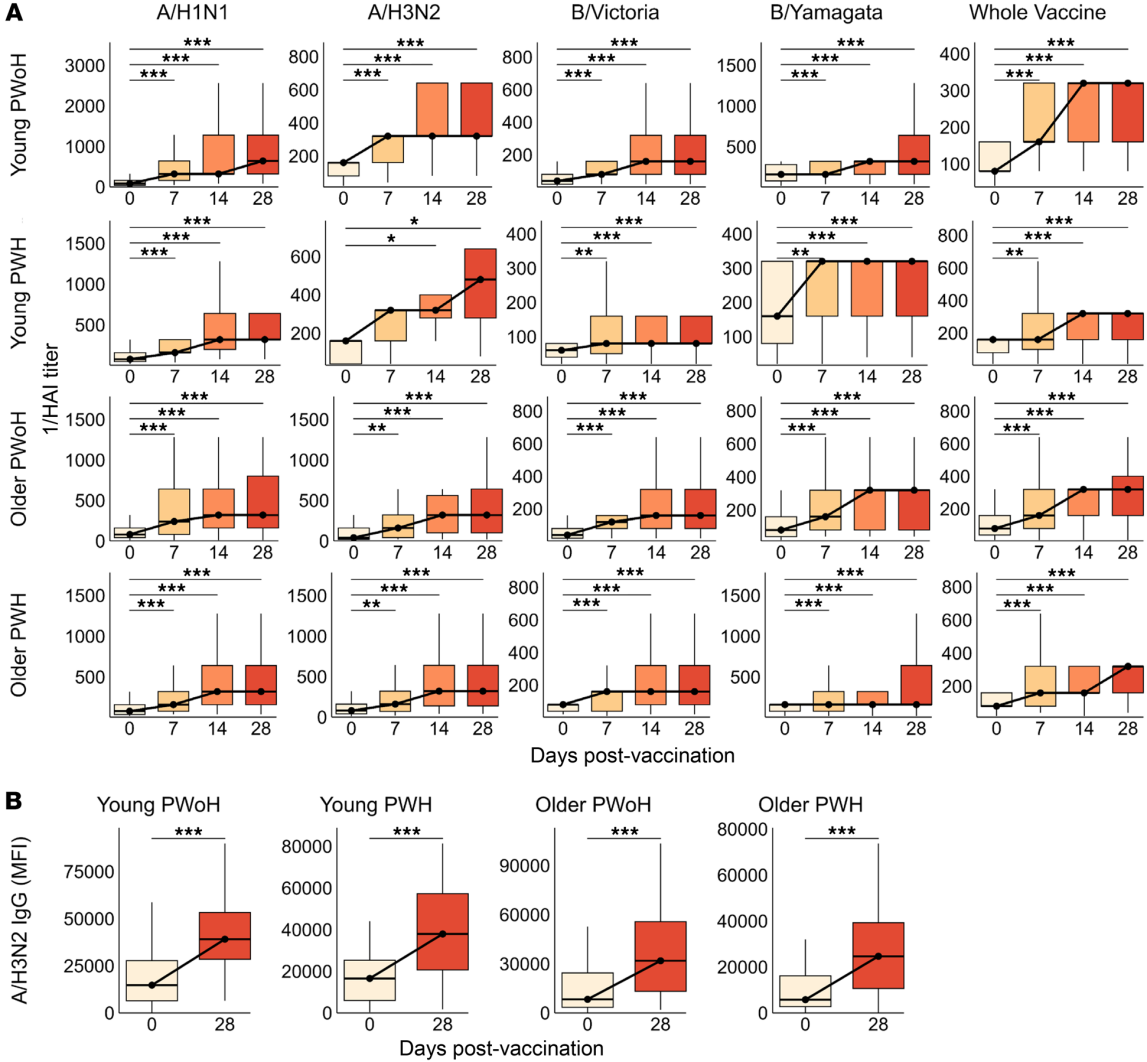
HAI responses rise sharply in the first month after standard-dose influenza vaccination, independent of age group and HIV status. (**A**) HAI titer against A/H1N1, B/Victoria, B/Yamagata, and whole vaccine antigens were assessed in young PWoH (*n* = 42, top row), young PWH (*n* = 30, second row from top), older PWoH (*n* = 60, second row from bottom), and older PWH (*n* = 51, bottom row) at 0, 7, 14, and 28 dpv (standard dose). A/H3N2 HAI titers were available for a subset of participants. (**B**) A/H3N2 HA-specific IgG (MFI) at 0 versus 28 days after standard-dose vaccination; young PWoH (*n* = 50), young PWH (*n* = 36), older PWoH (*n* = 68), and older PWH (*n* = 65). Box plots display the median and IQR, with whiskers extending to 1.5 × IQR and outliers excluded. *P* values are from Wilcoxon signed-rank tests for the 7–28 versus 0 dpv time points, adjusted for multiple comparisons by the Benjamini-Hochberg method. *P < 0.05; **P < 0.01; ***P < 0.001.

HAI titers against individual and whole vaccine antigens increased as early as 7 dpv and generally peaked by 14 dpv, at which point all participant groups exhibited significantly above-baseline responses to all vaccine antigens (Figure 2A and Supplemental Table 3). Given the limited A/H3N2 HAI dataset, we sought to infer seroprotection against this strain based on A/H3N2 HA-specific IgG levels. We observed a moderately strong correlation between A/H3N2 HAI titer and A/H3N2-specific IgG levels at 28 dpv (*r* = 0.58 [95% CI: 0.44–0.69], *P* < 0.001, Supplemental Figure 2). A/H3N2 HA-specific IgG levels markedly increased from 0 to 28 dpv across the 4 groups (Figure 2B). Similarly, HA IgG specific to A/H1N1, B/Victoria, and B/Yamagata antigens universally increased from 0 to 28 days after standard-dose vaccination, across all groups (Supplemental Figure 3).

**Figure 3:**
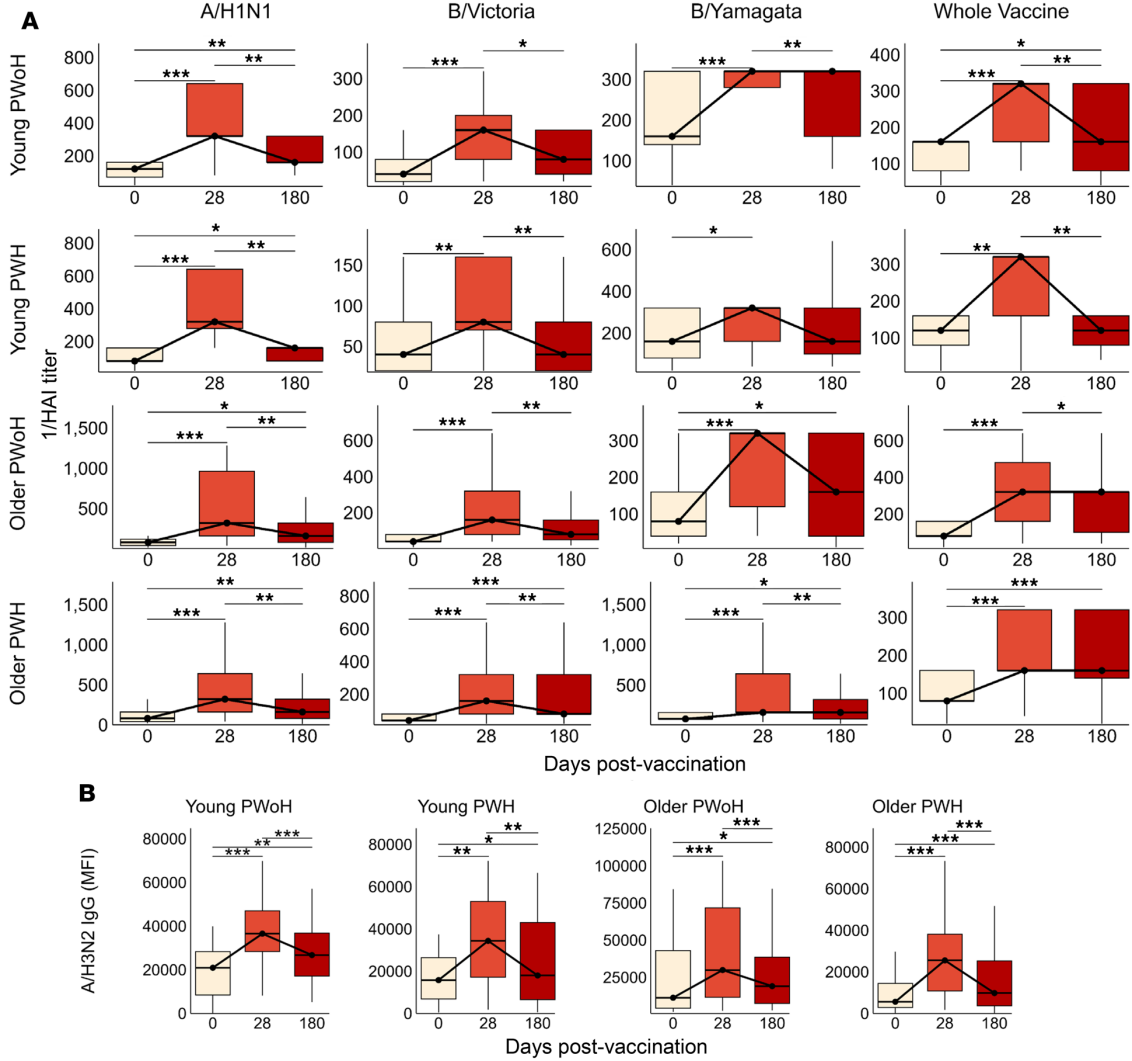
Seroprotective responses wane in the first 6 months after standard-dose influenza vaccination. In a subset of participants who returned for follow-up at 180 dpv after standard-dose influenza vaccination, antigen-specific serum responses were measured and compared with baseline (0 dpv) and peak (28 dpv) levels. (**A**) HAI titer against A/H1N1, B/Victoria, B/Yamagata, and whole vaccine for young PWoH (*n* = 20), young PWH (*n* = 18), older PWoH (*n* = 22), and older PWH (*n* = 44). (**B**) A/H3N2 HA-specific IgG levels (MFI); young PWoH, *n* = 31; young PWH, *n* = 20; older PWoH, *n* = 35; older PWH, *n* = 49. Box plots display the median and IQR, with whiskers extending to 1.5 × IQR and outliers excluded. *P* values are from Wilcoxon signed-rank tests between the 3 time points, adjusted for multiple comparisons by the Benjamini-Hochberg method. *P < 0.05; **P < 0.01; ***P < 0.001; unlabeled comparisons are not significant.

We next assessed the durability of vaccine responses in a subset of participants who provided both 0 and 28 dpv samples and who returned for follow-up at 6 months (180 days) after standard-dose vaccination (young PWoH, *n* = 20; young PWH, *n* = 18; older PWoH, *n* = 22; and older PWH, *n* = 44). From 28 to 180 dpv, HAI titers against A/H1N1 and B/Victoria declined significantly across all groups. Titers against B/Yamagata declined among young PWoH and older PWH, and whole vaccine titers declined across all groups except older PWH (Figure 3A). Small reductions in the proportion of individuals with seroprotective HAI titers against A/H1N1, B/Victoria, and B/Yamagata antigens were also evident across the groups (Supplemental Table 4) Despite the pattern of decline, titers at 180 dpv remained significantly elevated above prevaccination levels for A/H1N1 (all groups), B/Victoria (older PWH only), B/Yamagata (older PWoH and older PWH), and whole vaccine (young PWoH and older PWH). Levels of A/H3N2-specific IgG declined sharply from 28 to 180 dpv but remained elevated above baseline levels at 180 dpv across the 4 groups (Figure 3B). Similarly, IgG specific to A/H1N1, B/Victoria, and B/Yamagata strains declined significantly from 28 to 180 dpv. Above-baseline A/H1N1-specific IgG levels persisted among all groups except older PWH, and B/Victoria-specific and B/Yamagata-specific IgG levels returned to baseline at 180 dpv across the 4 groups (Supplemental Figure 3).

### Aging with HIV infection is associated with diminished influenza A/H1N1 responses.

Given the heterogeneous composition of the 4 groups, we conducted a multivariate analysis to determine the effects of aging and HIV infection on vaccine responses while controlling for the effects of self-reported demographic variables, self-reported influenza vaccination history, and prevaccination serological responses, which differed between the groups. Self-reported influenza vaccination at any time point prior to study enrollment was associated with reduced A/H1N1-specific (β = –8512, *P* = 0.005), B/Victoria-specific (β = –9357, *P* < 0.001), and B/Yamagata-specific (β = –11,234, *P* < 0.001) IgG levels and reduced HAI titer against B/Yamagata (OR = 0.36 [0.19–0.68], *P* = 0.002). Participant sex was not associated with HAI titer or HA-specific IgG responses to standard-dose vaccination (Supplemental Table 5).

**Figure 4:**
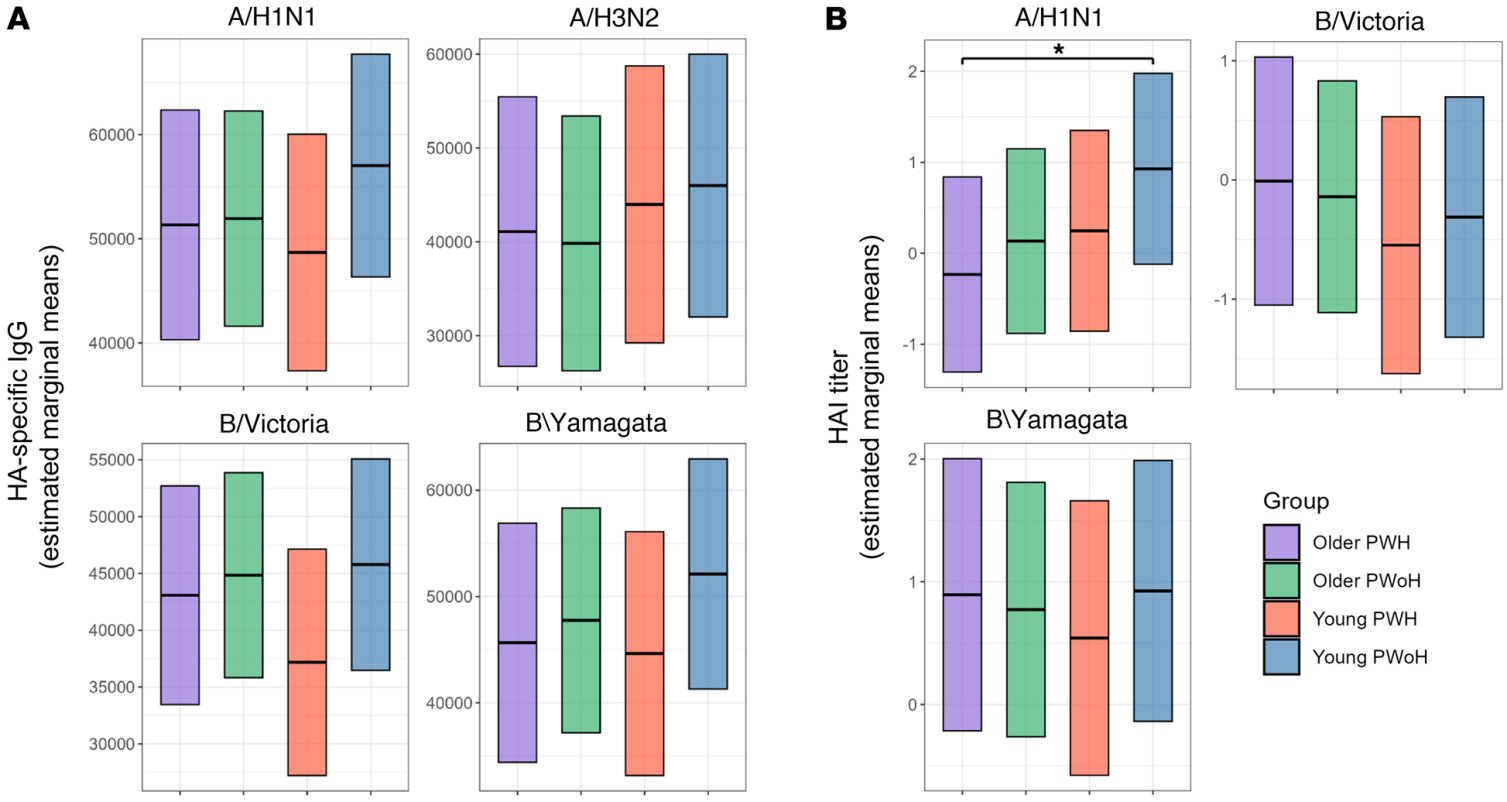
Serum A/H1N1 responses are reduced among older PWH at 28 days after standard-dose influenza vaccination. Multivariate comparison of (**A**) strain HA-specific IgG levels and (**B**) influenza HAI titer at 28 days after standard-dose vaccination for young PWoH (*n* = 50, blue), young PWH (*n* = 35, red), older PWoH (*n* = 68, green), and older PWH (*n* = 63–64, purple). Box plots display estimated marginal means from a linear regression model controlling for the effects of baseline vaccine responses, previous influenza vaccine history, and demographic variables. *P < 0.05.

Regression analysis of HAI responses at 28 dpv revealed no differences between the groups in HA-specific IgG levels or HAI titer against B/Victoria or B/Yamagata antigens. In contrast, older PWH showed markedly reduced HAI titer against A/H1N1 compared with young PWoH (OR = 0.31, *P* = 0.021), whereas a similar impairment was not observed among young PWH or older PWoH (Figure 4). Regression of age group and HIV status as individual covariates revealed a significant impact of older age on A/H1N1 HAI responses (OR = 0.53, *P* = 0.016), and the presence of HIV infection was independently associated with trends of lower A/H1N1 HAI titer (OR: 0.61, *P* = 0.055) and B/Victoria IgG (β= –4332, *P* = 0.051) responses (Supplemental Table 6). Consistent with the multivariate age group findings, univariate correlational analysis revealed negative associations between chronological age and A/H1N1 HAI titer at 28 dpv among PWoH (ρ = –0.18, *P* = 0.048) and PWH (ρ = –0.18, *P* = 0.075), which were not evident at the prevaccination time point (Supplemental Figures 4 and 5).

In summary, all groups responded well to standard-dose influenza vaccination, and significant reductions in the magnitude (HAI titer) and durability (HA-specific IgG) of seroprotective influenza A/H1N1-specific responses were observed among older PWH.

### High-dose vaccination improves influenza B/Victoria and A/H3N2 IgG responses of older individuals regardless of HIV status, and A/H1N1-specific serological responses are unchanged.

Among participants who received standard-dose vaccination, 123 returned for high-dose vaccination in the consecutive season (young PWoH, *n* = 17; young PWH, *n* = 16; older PWoH, *n* = 49; older PWH, *n* = 41). Among high-dose recipients, participant groups were well balanced in terms of demographic data and pre-study influenza vaccination (Table 2).

**Figure 5:**
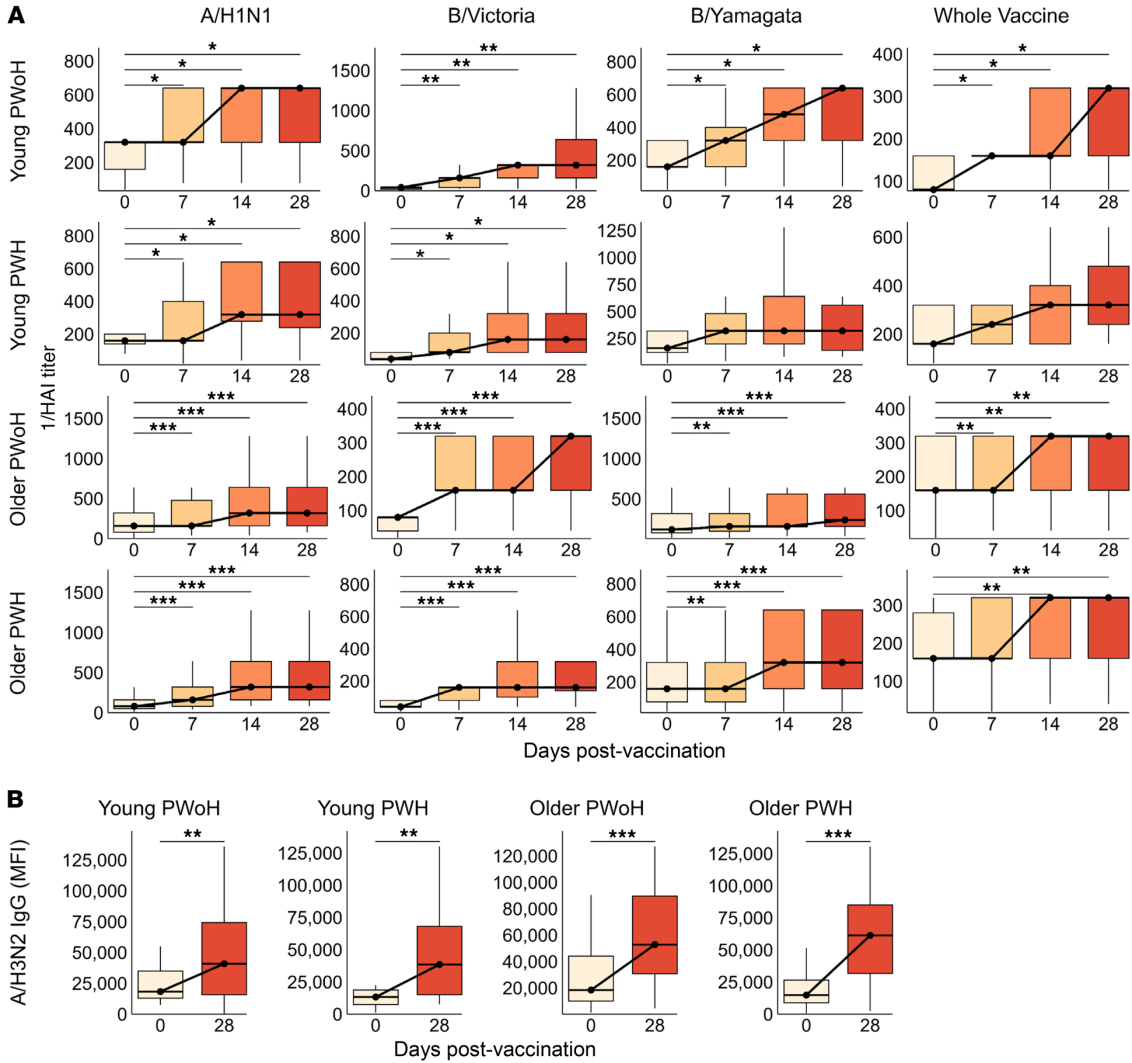
Seroprotective responses rise sharply in the first month after high-dose influenza vaccination. Analysis includes participants who attended all follow-up visits in the first month after high-dose vaccination. (**A**) HAI titer against A/H1N1, B/Victoria, B/Yamagata, and whole vaccine antigens were assessed in young PWoH (*n* = 13, top row), young PWH (*n* = 11, second row from top), older PWoH (*n* = 41, second row from bottom), and older PWH (*n* = 36, bottom row) at 0, 7, 14, and 28 dpv (high-dose). B/Yamagata HAI titers were not assessed in participants administered trivalent vaccine during the 2024–2025 flu season (young PWoH, *n* = 2; young PWH, *n* = 2; older PWoH, *n* = 12; older PWH, *n* = 10). (**B**) A/H3N2 HA-specific IgG (MFI) at 0 versus 28 days after high-dose vaccination; young PWoH (*n* = 17), young PWH (*n* = 15), older PWoH (*n* = 47), and older PWH (*n* = 40). Box plots display the median and IQR, with whiskers extending to 1.5 × IQR. *P* values are from Wilcoxon signed-rank tests for the 7–28 dpv versus 0 dpv time points, adjusted for multiple comparisons by the Benjamini-Hochberg method. *P < 0.05; **P < 0.01; ***P < 0.001.

At the high-dose season baseline, a similar frequency of study participants had seroprotective HAI titer levels against A/H1N1 (95% vs. 93%, *P* = 0.50), B/Victoria (82% vs. 73%, *P* = 0.051), and B/Yamagata (90% vs. 94%, *P* = 0.25) compared with at the time point of study entry (standard dose, 0 dpv), and the frequency of seroprotective titers at the baseline time point of the high-dose season did not differ between the groups (Supplemental Table 7). As observed after standard-dose vaccination, high-dose vaccination drove marked HAI and HA-specific IgG responses as early as 7 dpv. HAI titers against A/H1N1 and B/Victoria, along with A/H3N2-specific IgG, significantly increased from 0 to 28 dpv across the 4 groups, and whole vaccine HAI titers increased in all groups except young PWH (Figure 5). At 28 days after high-dose vaccination, seroprotective titers against A/H1N1, B/Victoria, and B/Yamagata were present in nearly all participants (Supplemental Table 8).

Similar to the standard-dose analyses, multivariate analyses of high-dose responses at 28 dpv revealed no effect of sex on HAI titer or HA-specific IgG responses (Supplemental Table 9). After controlling for covariates, we observed higher B/Yamagata and lower B/Victoria responses among young PWoH compared with older PWoH, and impairment in A/H1N1 HAI titer among older PWH was not evident after high-dose vaccination (Supplemental Figure 6). These differences appeared to be driven largely by age group, rather than HIV status (Supplemental Table 10).

**Figure 6:**
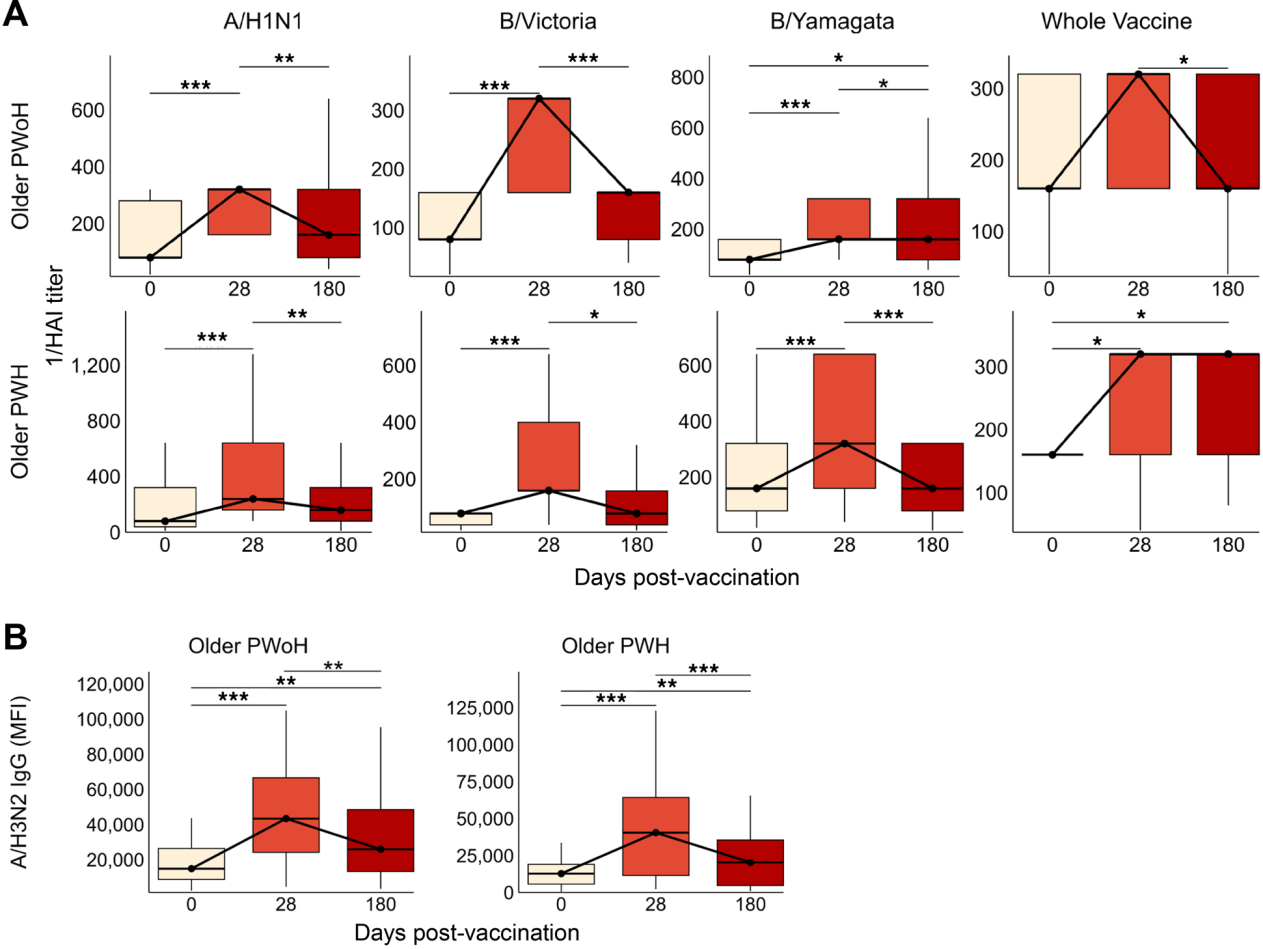
Serologic responses decline in the first 6 months after high-dose influenza vaccination. In a subset of participants who returned for follow-up at 180 dpv (high-dose vaccine), antigen-specific responses were measured and compared with baseline (0 dpv) and peak (28 dpv) levels. (**A**) HAI titer against A/H1N1, B/Victoria, B/Yamagata, and whole vaccine in older PWoH (*n* = 25) and older PWH (*n* = 24). B/Yamagata HAI titers were not assessed in participants administered trivalent vaccine during the 2024–2025 influenza season (older PWoH, *n* = 12; older PWH, *n* = 10). (**B**) A/H3N2-specific IgG (MFI) at 0, 28, and 180 dpv; older PWoH, *n* = 26; older PWH, *n* = 25. Box plots display the median and IQR, with whiskers extending to 1.5 × IQR and outliers excluded. *P* values are from Wilcoxon signed-rank tests between the 3 time points, adjusted for multiple comparisons by the Benjamini-Hochberg method. *P < 0.05; **P < 0.01; ***P < 0.001; unlabeled comparisons are not significant.

As with standard-dose vaccination, we evaluated longitudinal responses to influenza antigens in a subset of participants who provided samples at 0, 28, and 180 days after high-dose vaccination. Sample sizes for longitudinal comparisons were sufficiently large for older PWoH (*n* = 25) and older PWH (*n* = 24), but too few young PWoH or young PWH provided longitudinal samples during the high-dose season to reliably gauge the durability of responses. Among both of the older groups, HAI titers and HA IgG specific to all vaccine components significantly declined from 28 to 180 dpv. At 180 days after high-dose vaccination, HAI titers against A/H1N1, B/Victoria, and whole vaccine returned to baseline levels for both groups, and elevated IgG responses were maintained for A/H3N2 (older PWoH and older PWH) and B/Victoria (older PWoH only) antigens (Figure 6, Supplemental Figure 7, and Supplemental Table 11).

**Figure 7:**
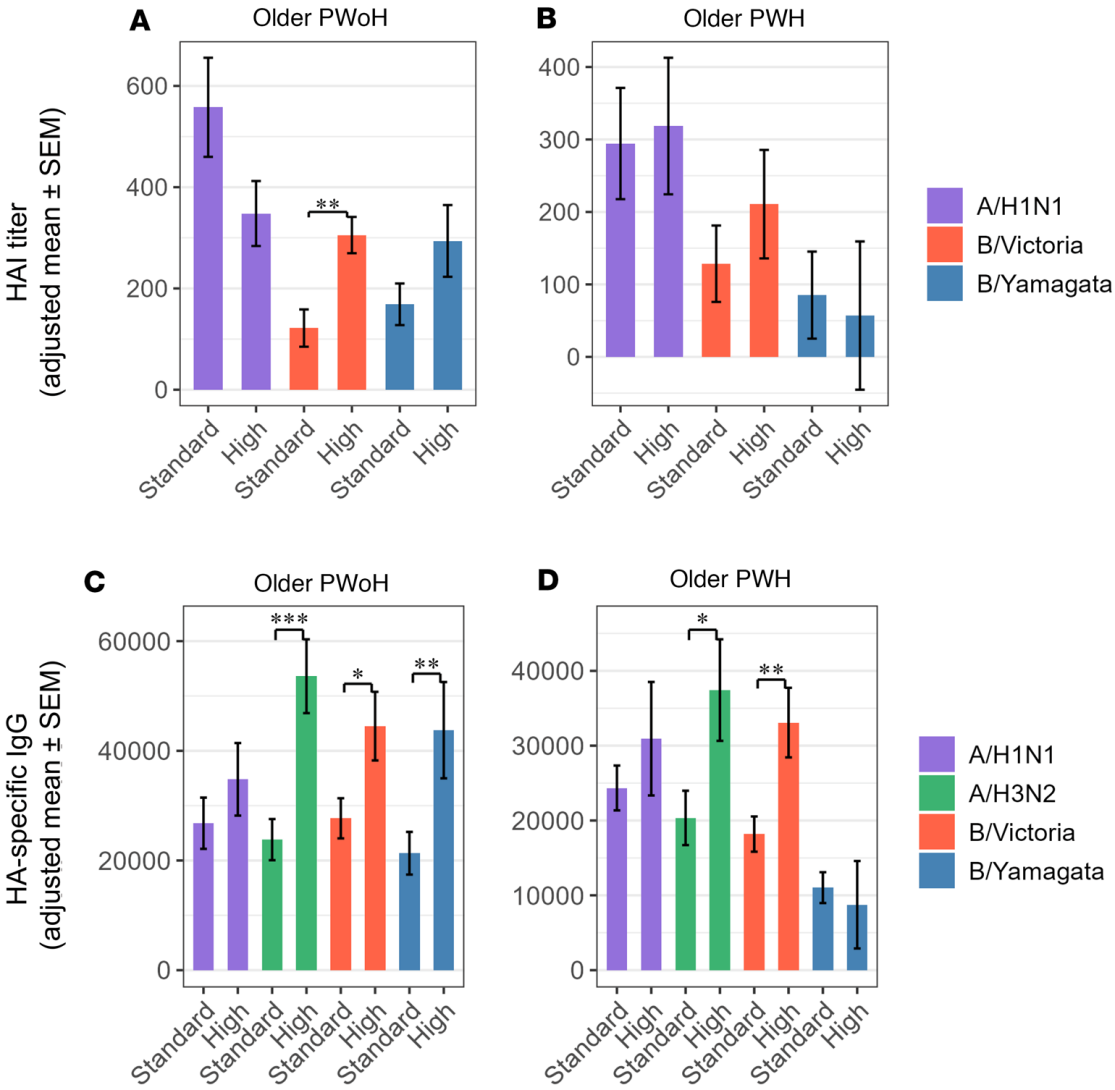
High-dose influenza vaccination improves A/H3N2-specific and B/Victoria-specific IgG responses of older people with and without HIV. Bar graphs depict estimated marginal means (EMMs) and standard errors of high-dose (“High”) versus standard-dose (“Standard”) influenza vaccine responses at 28 dpv. EMMs are from a mixed-effects model controlling for baseline (prevaccination) immune responses. (**A** and **B**) HAI titer against A/H1N1, B/Victoria (older PWoH, *n* = 40; older PWH, *n* = 30), and B/Yamagata (older PWoH, *n* = 32; older PWH, *n* = 26). (**C** and **D**) HA IgG specific to A/H1N1, A/H3N2, B/Victoria (older PWoH, *n* = 40; older PWH, *n* = 32) and B/Yamagata (older PWoH, *n* = 32; older PWH, *n* = 28). *P < 0.05; **P < 0.01; ***P < 0.001.

Given the number of participants who provided both 0 and 28 dpv serum samples for consecutive standard- and high-dose influenza seasons, we determined that regression analyses of high- versus standard-dose responses (evaluating the effect of vaccine dose while controlling for prevaccination immune responses) were sufficiently powered for both of the older groups (older PWoH, *n* = 40 and older PWH, *n* = 30). Among older PWoH and older PWH alike, IgG responses specific to A/H3N2 and B/Victoria antigens were significantly greater at 28 dpv for the high-dose versus standard-dose influenza vaccination. Improvements in B/Victoria HAI responses were observed among older PWoH but not older PWH, and neither IgG nor HAI responses specific to A/H1N1 differed between the 2 doses in either group. Conversely, we observed a nonsignificant trend of lower A/H1N1 HAI responses among older PWoH after high-dose versus standard-dose vaccination (*P* = 0.08, Figure 7).

In an exploratory analysis, we considered the effects of vaccine dose on immune responses of the combined younger cohorts (young PWoH and young PWH), providing sufficient power (*n* = 24–27) to detect interseasonal differences. Similar to what was observed among the older groups, vaccine dose was not associated with differences in A/H1N1-specific responses among the younger cohort, whereas B/Victoria-specific IgG levels were improved after high-dose vaccination. In contrast, A/H3N2-specific IgG responses were not improved among younger participants after high-dose vaccination, as was observed among the older cohorts, and prevaccination A/H3N2-specific responses were generally higher among the young participants (Supplemental Figure 8).

### High-dose influenza vaccination improves CD4^+^ T cell antigen-specific responses among older PWH.

To examine cellular mechanisms underlying influenza vaccine responses in aging and HIV, we assessed CD4^+^ and CD8^+^ T cell immunity in 0 and 14 dpv samples in a subset of participants (*n* = 59) selected from the larger cohort based on matching of group, age, and sex characteristics (Table 3). Prior influenza vaccination frequency was similar across the participant groups selected for cell-based analyses. An activation-induced marker (AIM) assay using CD69/CD40L (CD4^+^) or CD69/CD137 (CD8^+^) was used to evaluate total memory CD4^+^, peripheral T follicular helper (pTfh), and CD8^+^ T cell responses (see Supplemental Figure 9 for gating strategy and representative flow cytometry plots), as previously described (28).

**Figure 8:**
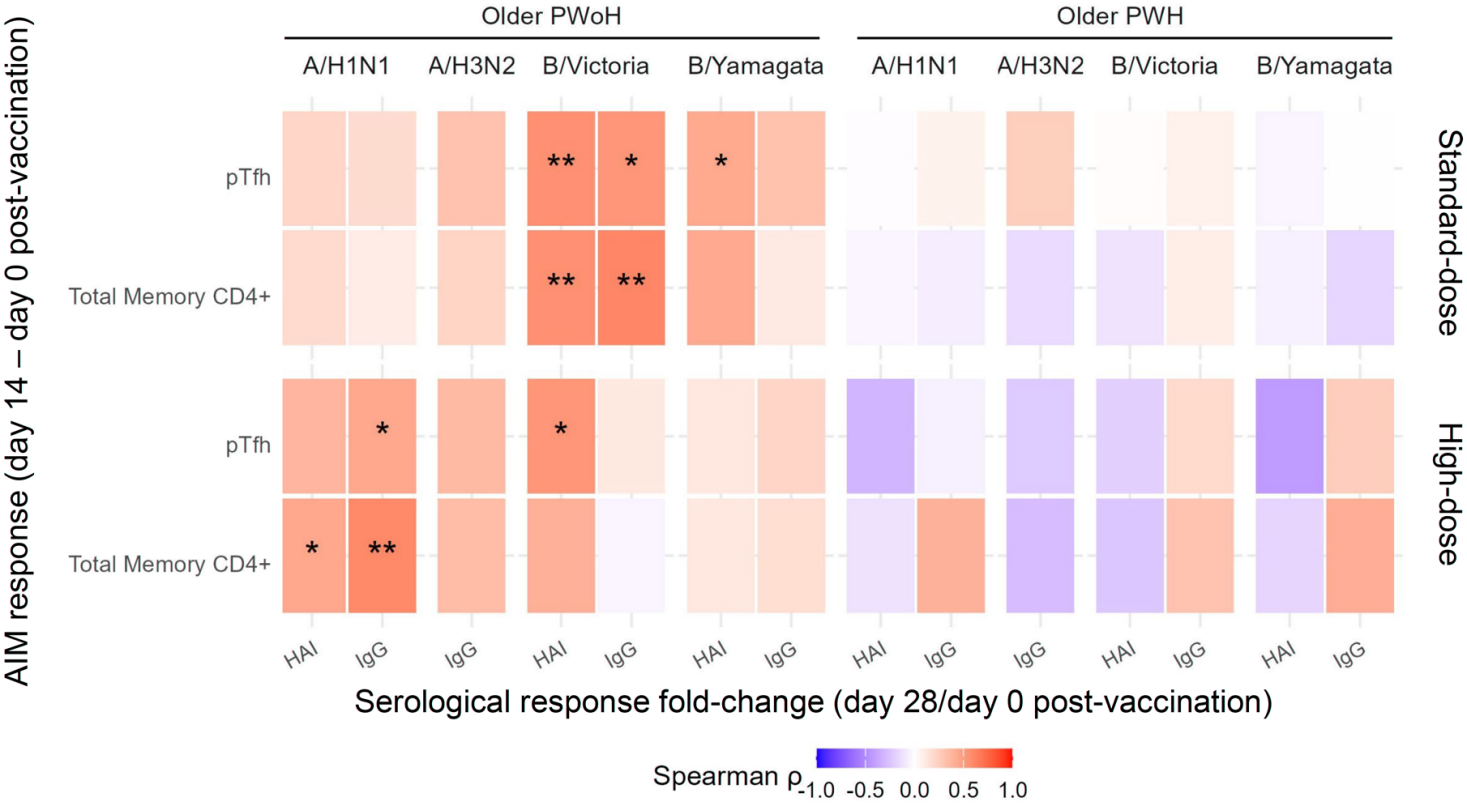
CD4^+^ T cell and pTfh AIM responses correlate with influenza seroprotection among older adults without, but not with, HIV. Heatmap showing Spearman’s correlations between AIM responses in peripheral T follicular helper (pTfh) cells and total memory CD4^+^ T cells, and serological vaccine responses in older PWoH (*n* = 20) and older PWH (*n* = 20). AIM responses were calculated as the media-adjusted, batched-normalized frequencies of CD69^+^CD40L^+^ cells at day 14 after vaccination relative to baseline (day 0). Serological responses were quantified as HAI titer or HA-specific IgG fold-change from day 0 to day 28, after standard- or high-dose influenza vaccination. *P < 0.05; **P < 0.01; unlabeled correlations are not significant.

Among older PWoH, the absolute change in B/Victoria-specific CD4^+^ T cell responses from 0 to 14 dpv (Δ14–0 dpv) correlated with serological responses (28/0 dpv fold-change) after both standard- and high-dose vaccination, whereas A/H1N1-specific CD4^+^ T cell responses correlated with serological responses only after high-dose vaccination. In contrast, no correlations were observed between CD4^+^ T cell AIM and antibody responses among older PWH after either vaccine dose (Figure 8). Correlations among younger participants were nonsignificant but generally limited by small sample sizes (*n* = 9–10 per group, Supplemental Figure 10).

**Figure 9:**
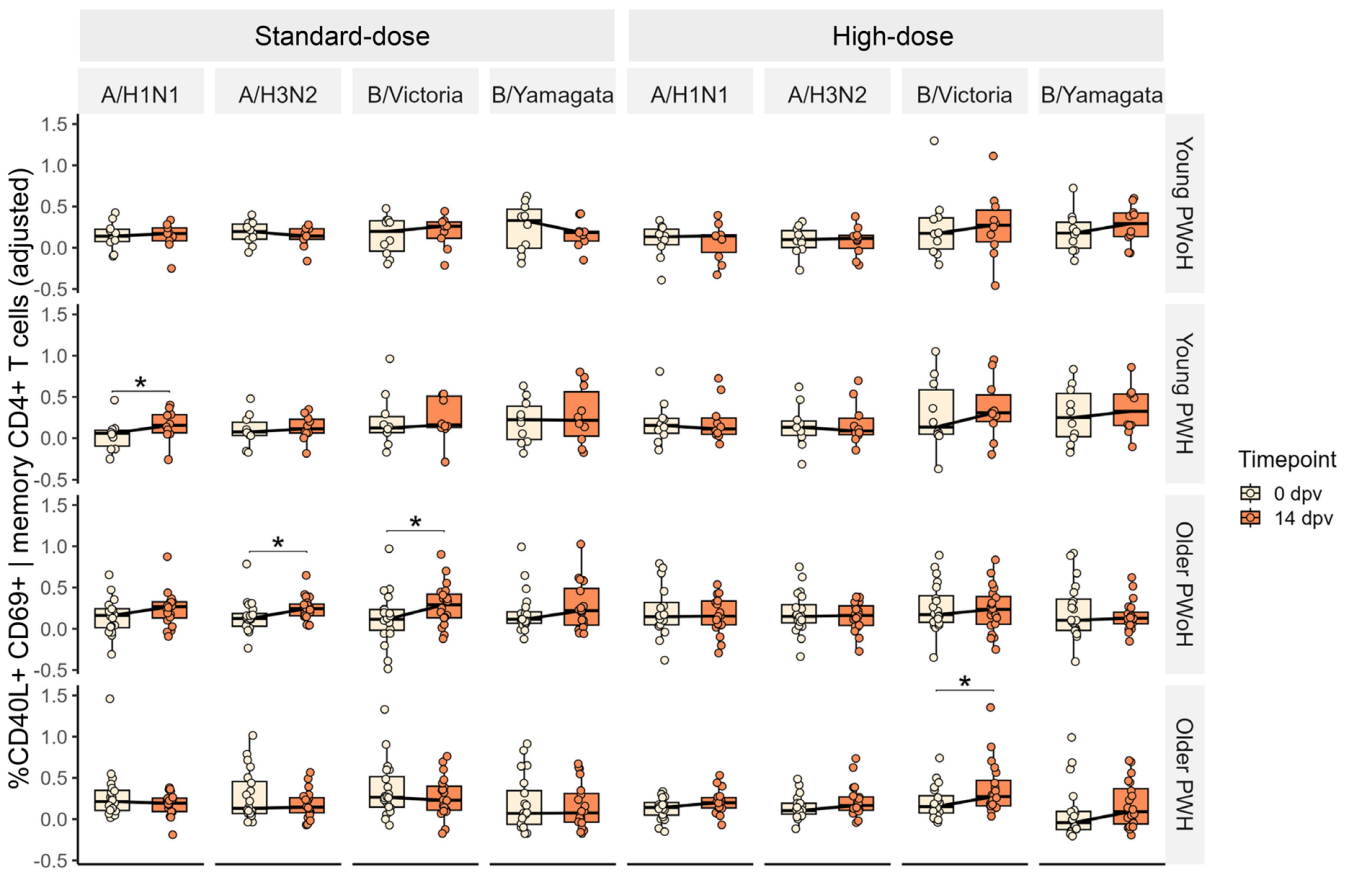
Memory CD4^+^ T cell antigen-induced marker responses after standard- and high-dose influenza vaccination. Media-adjusted, batch-normalized frequencies of CD40L^+^CD69^+^CD45RO^+^ memory CD4^+^ T cells are shown at 0 and 14 dpv. AIM responses were assessed in young PWoH (*n* = 9), young PWH (*n* = 10), older PWoH (*n* = 20), and older PWH (*n* = 20) after 12-hour stimulation of PBMCs with media or influenza antigens matched to the participants’ particular seasonal influenza vaccine strains. *P* values are from Wilcoxon signed-rank tests; *P < 0.05.

From 0 to 14 days after standard-dose vaccination, A/H3N2- and B/Victoria-specific total memory CD4^+^ T cells increased in older PWoH but not older PWH (Figure 9). After high-dose vaccination, older PWH showed significant responses only to B/Victoria, and significant changes were no longer observed among older PWoH. CD4^+^ pTfh and CD8^+^ T cell AIM responses did not increase among older PWH or older PWoH after either dose (Supplemental Figures 11 and 12).

**Figure 10:**
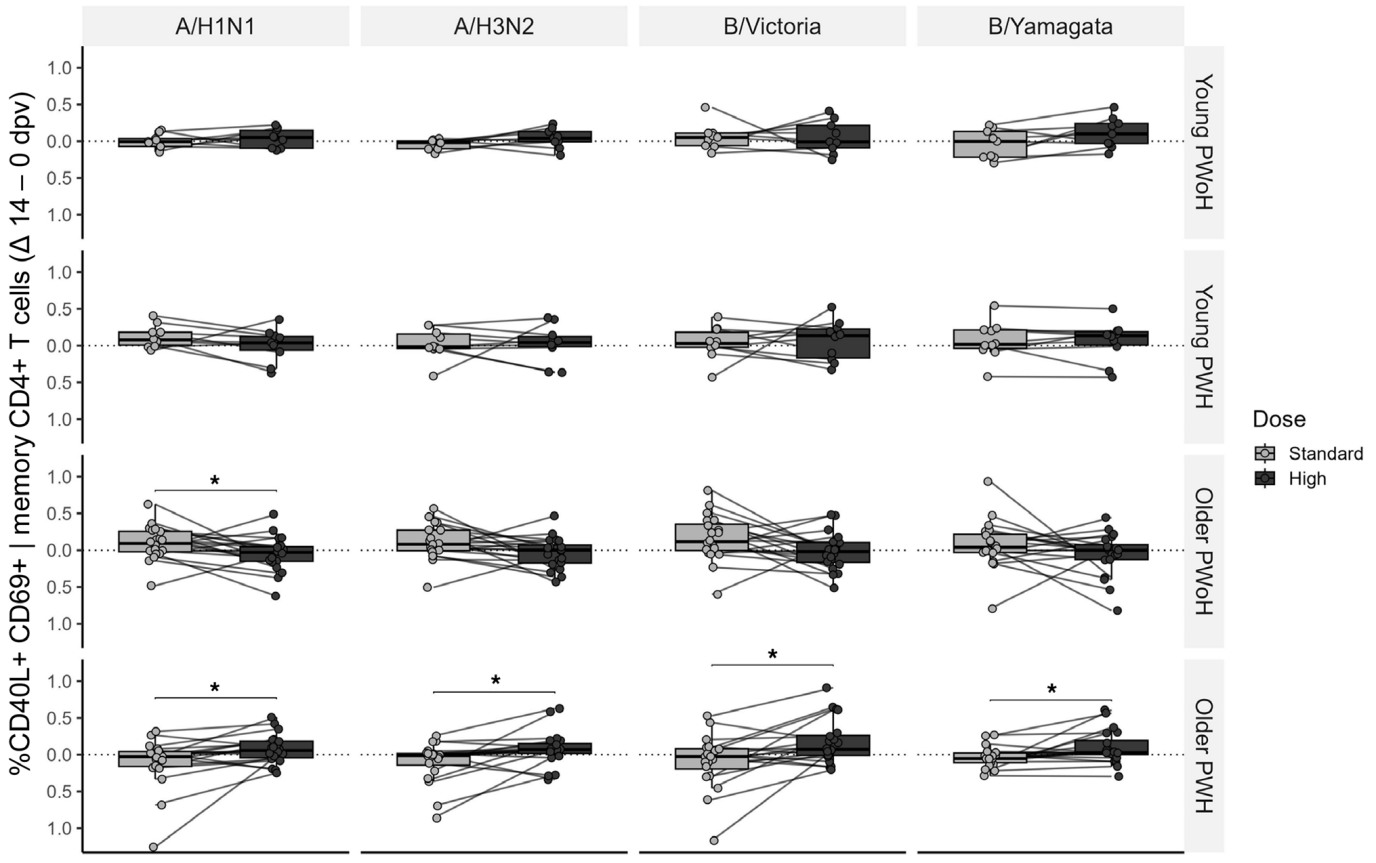
Change in memory CD4^+^ T cell antigen-induced marker responses after standard- versus high-dose influenza vaccination. Media-adjusted, batch-normalized frequencies of CD40L^+^CD69^+^ CD45RO^+^ memory CD4^+^ T cells were measured at 0 and 14 dpv (standard- and high-dose), and the change (Δ14–0 dpv) is plotted and compared between the 2 seasons. AIM responses were assessed in young PWoH (*n* = 9), young PWH (*n* = 10), older PWoH (*n* = 20), and older PWH (*n* = 20) after 12-hour stimulation of PBMCs with media or influenza antigens matched to the participants’ seasonal vaccine strain. *P* values are from Wilcoxon signed-rank tests; *P < 0.05.

To evaluate the cellular-level benefit of the high-dose vaccine, we compared the absolute change in response (Δ14–0 dpv) between seasons. Among older PWH, high-dose vaccination elicited greater total memory CD4^+^ T cell responses to A/H1N1, A/H3N2, B/Victoria, and B/Yamagata antigens compared with standard-dose vaccination (Figure 10). Among older PWoH, high-dose total memory CD4^+^ T cell and pTfh responses were either unchanged or reduced (A/H1N1), and CD8^+^ T cell responses did not differ between the 2 vaccine doses in any group (Supplemental Figures 13 and 14).

## Discussion

On a global scale, annual influenza outbreaks cause up to 5 million severe infections and 650,000 deaths, disproportionately burdening people over the age of 65 years (7, 29). Influenza vaccination, although considered the mainstay of disease prevention, currently has several limitations, including lower efficacy in seasons where vaccine antigen mismatch occurs and waning protection in older adults and people with compromised immune systems (12, 30, 31). High-dose vaccination, containing 4 times more antigen than the standard dose, attempts to overcome reduced vaccine efficacy and higher disease morbidity in older individuals. Generally, head-to-head comparisons of high- versus standard-dose vaccine effectiveness in people aged 65 years or older have found a slight reduction in infection and severe influenza outcomes among people who receive high-dose vaccination, although not all comparisons reached statistical significance (15, 16, 32). Here, we report distinct immune benefits of high- over standard-dose influenza vaccination in older adults with HIV across back-to-back influenza seasons, while also identifying gaps that novel vaccination strategies may attempt to address.

Among the population of PWH, which is rapidly aging and approaching 40 million individuals worldwide, it is well established that influenza vaccination is both safe and effective (33). Yet, few studies have directly estimated the benefit of high-dose vaccination in this population, and current guidelines do not directly address the situation of virally suppressed PWH. Viral suppression with ART greatly improves immune function and reduces the likelihood of severe disease with influenza or other infections. However, cell-intrinsic defects persist, resulting in virally suppressed PWH continuing to mount weaker responses to vaccination (19–21, 34, 35). Whether high-dose influenza vaccination might overcome these deficits is not known. In the present study, we found evidence of potent immune responses to standard- and high-dose vaccination among older PWH, which waned significantly from 1 to 6 months after vaccination. At 1 month after vaccination, older PWH had significantly elevated HAI titers and HA-specific IgG against influenza A and B strains, regardless of dose, and titers at 1:40 or higher were nearly universal. Still, in well-controlled multivariate analyses, older PWH were observed to have reduced influenza vaccine responses compared with younger participants, whereas no similar impairment was observed among older PWoH. The lack of an expected age-associated reduction in serum responses among older PWoH compared with young PWoH may be a product of sample size limitations in the rigorously controlled multivariate analyses and/or differences in pre-study influenza vaccination and infection history. Indeed, multivariate analysis assessing age group and HIV status as separate covariates revealed independent, negative effects of both older age and HIV infection on A/H1N1 HAI responses. These findings are in line with our previous study on standard-dose influenza vaccine responses, in which we observed a cumulative effect of aging and HIV infection in suppressing A/H1N1-specific vaccine responses (19).

Compared with the previous year’s standard-dose vaccination, high-dose vaccination significantly improved A/H3N2- and B/Victoria-specific antibody responses among older PWoH and PWH alike. Among older PWoH, these improvements were observed at the level of both HAI titer and HA-specific IgG, whereas improvements among older PWH were limited to HA-specific IgG. A/H3N2-specific IgG, in turn, correlated moderately well with A/H3N2 HAI titers in a subset of participants vaccinated during the 2020–2021 and 2021–2022 seasons, suggesting that it may serve as a surrogate of seroprotection. Influenza B infections, now caused exclusively by B/Victoria-lineage viruses, have historically accounted for 10%–20% of annual influenza infections and are increasingly recognized to cause significant morbidity and mortality in older people; A/H3N2 comprises up to half of seasonal influenza infections and has significant pandemic potential (11, 36). In agreement with findings of lower relative efficacy of the high-dose vaccine against protocol-defined influenza-like illness caused by A/H1N1 versus A/H3N2 or B strains and reports of reduced relative effectiveness of high-dose vaccination in A/H1N1-dominant seasons, we failed to observe improved A/H1N1-specific serological responses among older participants with or without HIV after high-dose vaccination (15, 37). In fact, we observed a trend of lower A/H1N1 HAI responses among older PWoH after high-dose versus standard-dose vaccination, and self-reported pre-study influenza vaccination was associated with significantly diminished A/H1N1-specific IgG responses, consistent with a recent report that repeated vaccination is associated with lower influenza A–specific postvaccination titers (38). At the cellular level, we observed reduced A/H1N1-specific memory CD4^+^ T cell AIM responses after high-dose vaccination, which may partially explain the serological findings. High-dose vaccination was associated with more robust memory CD4^+^ T cell responses to A/H1N1, A/H3N2, B/Victoria, and B/Yamagata antigens among older PWH compared with standard-dose vaccination; however, these increases did not correlate significantly with antigen-specific serological responses, suggesting a potential cell-intrinsic defect limiting effective antibody production. In contrast, we observed no induction of CD8^+^ T cell AIM responses after either standard- or high-dose vaccination and no significant differences between the two doses, consistent with prior studies showing ineffective CD8^+^ T cell stimulation from inactivated vaccines compared with live attenuated vaccines or natural infection (39, 40).

Our findings suggest that the immune response to influenza vaccination is influenced by both antigen concentration and immunodominance, which is altered by age and HIV status. In our study, older individuals had higher responses to B/Victoria antigens both at baseline and after vaccination, with a particularly wide advantage compared with young individuals after high-dose vaccination. Understanding the immunological differences between the antigen-specific responses may guide vaccine design, particularly in the use of adjuvants or strategies to enhance the immunogenicity of A/H1N1 components. A fixed dose, as currently used, may be insufficient to effectively boost the immune response; instead, increasing the antigen dose for the less immunodominant antigens or reducing the highly immunodominant antigen dose may yield a broader and more sustained response. In a recent study, subtype bias was overcome by covalently coupling HA antigens from different strains (41). Additionally, there is a high level of interest in developing a universal influenza vaccine, which could provide long-term protection against multiple strains (42).

In analyses on the durability of vaccine responses after standard-dose vaccination, we found evidence of persistently elevated responses at 180 dpv, which varied by antigen and group. HAI responses against A/H1N1-specific and A/H3N2-specific IgG remained at above-baseline levels through 6 months after vaccination across the groups, while B/Victoria responses persisted solely among older PWH. In contrast with HAI titer, older PWH did not maintain A/H1N1-specific IgG responses at 180 dpv, as was observed across the other groups. Discrepancies between HAI titer and quantitative HA-specific IgG have been reported previously (43) and may reflect their functional versus quantitative nature, whereby high levels of antibodies with poor neutralizing capacity fail to produce an HAI response. On the other hand, minor increases in highly potent neutralizing antibodies may produce significant hemagglutination. After high-dose vaccination, older PWH again showed a waning of A/H1N1 and B/Victoria HAI and IgG responses, which returned to baseline levels by 6 months after vaccination. In the Northern hemisphere, annual influenza vaccination typically begins in August, and cases generally peak between December and March (44). Our results provide evidence that current vaccination regimens might not afford sustained protection against A/H1N1 infection later in the season, particularly among older PWH.

There are some notable limitations to our study. Importantly, since no group of participants received a standard-dose vaccine in consecutive seasons (i.e., standard dose followed by standard dose), we could not conclusively distinguish the effects of repeated vaccination from dose-specific effects. However, as more than 60% of participants reported past influenza vaccination, including nearly 90% of PWH, it is unlikely that standard-dose vaccination uniquely primed responses to high-dose vaccination. Further, we stringently controlled for baseline immune responses and pre-study vaccination in multivariate analyses. Crossover trials have previously been used to assess a “mix-and-match” COVID-19 vaccination strategy in which participants were administered a booster dose of the same or different formulation as their primary dose (45). By implementing a similar design, we were able to directly assess any additional benefit that consecutive high-dose vaccination conferred within our study cohorts. These conditions may recapitulate the situation of millions of vaccine-experienced older adults who “age into” current high-dose influenza vaccination guidelines each year. In terms of sample size, we were generally limited in the ability to robustly compare high-dose versus standard-dose responses among younger participants, including younger PWH, and multivariate models were underpowered to control for variables such as self-reported comorbidity or multimorbidity status. Vaccine responses were, however, uniformly high among the younger groups, with no significant differences detected between young PWH and PWoH. In the present study, which overlapped with the COVID-19 pandemic, we did not collect detailed data on COVID-19 infection or vaccination. Coadministration of COVID-19 and influenza vaccination is not associated with reduced immunogenicity compared with single-visit vaccination (46), and it is unknown whether COVID-19 infection itself could affect the magnitude or durability of influenza vaccination responses.

The difficulty in reliably detecting A/H3N2-specific HAI titers reflects the well-described impact of antigenic drift on HA-erythrocyte interactions, which can compromise hemagglutination-dependent assays (47). Although HA-specific IgG data provided some insight into A/H3N2-specific seroprotection, future studies may use alternative strategies to assess A/H3N2-specific neutralization among PWH after high-dose vaccination, such as imaging-based microneutralization (48). HAI titer and HA-specific IgG responses are correlates of protection against influenza, but our study did not assess clinical outcomes, including laboratory-confirmed influenza infection; as such, we cannot make a conclusive statement regarding the clinical benefit of a high-dose vaccine in older PWH. Furthermore, symptomatic or subclinical influenza infections occurring during the study period may have enhanced or prolonged persistence of participants’ serological responses (49). In addition to the readouts of HAI titer and HA-specific IgG, future studies on vaccine responses in aging PWH should consider additional measures, such as affinity/avidity and neutralization capacity, or measurement of antibody-secreting cells, to more completely capture the breadth of antibody responses from early to late postvaccination time points. Recent work has suggested that antibody-affinity maturation may influence the effectiveness of serum responses during repeated vaccination (50). Incorporating additional post-peak sampling time points (e.g., 60–120 dpv) would allow for a more precise characterization of immune response decay kinetics, as the current design, with only 28 and 180 dpv measurements, limits temporal resolution of the decline phase.

Finally, it is important to note that cell-based analyses, although insightful, were limited in scope and sample size. We did not directly assess CD4^+^ T cell subset (Th1, Th2, Th17) or Treg phenotype or function, which may play important roles in modulating vaccine effectiveness in aging and in chronic HIV infection. The lack of a measurable CD8^+^ T cell response, although consistent with previous reports on unadjuvanted, inactivated influenza vaccination (51), should be interpreted with caution given that CD8^+^ T cells process antigen in the context of MHC-I and may thus show minimal activation after whole peptide stimulation, as was performed in the present study. Further work is needed to more comprehensively interrogate the breadth of vaccine-specific T cell responses in older PWH and to determine how these responses relate to protection against clinical disease.

Ultimately, this study emphasizes the importance of including older PWH in influenza vaccination studies to assess the relative magnitude and durability of vaccine-derived protection. We provide evidence for improved serological and cellular responses within virally suppressed older PWH after high-dose vaccination while identifying the need to devise alternative strategies to generate stronger and longer-lived A/H1N1 responses and more potent cytotoxic T cell responses to enhance protection of this rapidly growing population with unique immune vulnerabilities.

## Methods

### Sex as a biological variable.

Our study examined male and female younger and older people with and without HIV. Multivariate analyses evaluated and controlled for the effects of sex on influenza vaccine responses.

### Setting and participants.

The current study was conducted during the influenza seasons of 2020–2021, 2021–2022, 2022–2023, 2023–2024, and 2024–2025. Participants provided written informed consent and were recruited at the University of Miami clinics into the HIV and Aging study cohort (NCT04487041). Participants were grouped by age as young (≤40 years) and older (≥60 years) PWoH or PWH. All PWH were on ART with a plasma viral load of less than 40 copies/mL for at least 1 year prior to study entry. Median duration of ART was 6.5 and 20.0 years for the young PWH and older PWH groups, respectively. Participants receiving hormone therapy, steroids, or immunosuppressant medications or who had diagnoses of malignancies or other non-HIV immunodeficiency disorders were excluded from the study. Additional information on the study protocol and selection criteria can be found at the ClinicalTrials.gov website.

At study entry, each participant received a single intramuscular standard-dose quadrivalent influenza vaccine (QIV; 15 μg HA per strain). During the 2021–2022 through 2024–2025 seasons, all administered vaccines were egg-based (Fluzone, Sanofi Pasteur; see Table 4 for vaccine composition). In the 2020–2021 season, the vaccine platform varied because of reduced availability of egg-based vaccine during the COVID-19 pandemic: 12 participants received cell-based vaccine (Flucelvax, Seqirus), 14 received Fluzone, and for 5 participants the vaccine product could not be confirmed. Peripheral venous blood was collected at prevaccination and postvaccination time points, including 7, 14, 28, and 180 dpv. In the consecutive influenza vaccination season (approximately 1 year after standard-dose vaccination), a subset of participants returned to receive a single Fluzone high-dose QIV (60 μg of HA antigen per strain), and blood was collected at the same pre- and postvaccination time points. Participants vaccinated during the 2024–2025 influenza season received trivalent vaccine (no B/Yamagata component). Participant phlebotomy, serum separation, and isolation of PBMCs by density gradient centrifugation were performed on the same day and stored at −80 °C (serum) or in a cryogenic liquid nitrogen freezer (PBMCs) until further use.

### Serologic responses to influenza vaccination.

Serologic responses to season-specific influenza whole vaccine, A/H1N1, A/H3N2, B/Victoria, and B/Yamagata antigens were measured by HAI assay (28) and by an HA-specific influenza antibody detection multiplex Luminex assay on serum from each of the previously described standard- and high-dose time points. The HAI assay was performed over 2 days; first, the serum was incubated with receptor-destroying enzyme overnight, then 2-fold serial dilutions of 1:10 diluted serum were incubated with recombinant influenza antigens (Sino Biological) for 1 hour. After incubation, 1:100 diluted chicken RBCs were added to each serum titration for at least 2 hours before reading results. Given that A/H3N2 HA antigen from the most recent influenza seasons was unable to effectively agglutinate RBCs, A/H3N2-specific HAI data could only be collected during the 2020–2021 and 2021–2022 seasons. For the HA Luminex assay, 1:200-diluted serum was incubated with HA-coupled xMAP beads (DiaSorin) on a shaker overnight at 4°C. Bead coupling was performed by a 2-step carbodiimide conjugation as previously described (52). PE-conjugated anti-human IgG secondary antibodies were added to the serum-bead mixture and incubated on a shaker for 1 hour. MFI of PE was read on the Flex MAP instrument (Thermo Fisher Scientific) and averaged across technical duplicates as a relative quantitative readout of the antigen-specific IgG level.

### Cellular responses to influenza vaccination.

Among participants who provided 0 and 14 dpv PBMC samples during both the standard- and high-dose seasons, a subset of 59 participants was selected for analysis of T cell responses to vaccination. Samples were processed using a previously described combined AIM/intracellular cytokine staining assay (28). For the present study, analyses were restricted to the AIM component, identifying antigen-responsive total CD45RO^+^ memory and CD45RO^+^CXCR5^+^ pTfh CD4^+^ T cells expressing CD40L and CD69 and antigen-responsive CD8^+^ T cells expressing CD137 and CD69. Participants were selected from the 4 groups with matching based on sex (younger PWoH/PWH, *n* = 9–10; older PWoH/PWH, *n* = 20). Cellular responses were assessed in cryopreserved PBMCs, which were thawed and rested for 3 hours at 37°C w/5% CO_2_ in R10 media (Gibco RPMI 1640 + 10% FBS + 100 U/mL penicillin-streptomycin + 2 mM l-glutamine) prior to stimulation. After resting, PBMCs (1 million per condition) were incubated with 2 μg/mL influenza antigen (A/H1N1, A/H3N2, B/Victoria, B/Yamagata) matched to the participants’ seasonal influenza vaccine or media in the presence of 1 μg/mL anti-CD28 costimulation (BD Biosciences) for 12 hours at 37°C w/5% CO_2_, with the addition of 0.4 mg/mL brefeldin A (MilliporeSigma) after 3 hours to block cytokine secretion. After stimulation, cells were resuspended in PBS containing 50 μL/mL Human TruStain FcX Receptor Blocking solution (BioLegend) and 0.5 μL/mL Live/Dead Fixable blue (Thermo Fisher Scientific) for 15 minutes at 4°C. Surface staining was performed in 100 μL PBS with 0.5% FBS containing the following antibodies: CD45-BV570, CD3-BUV395, CD4-BUV805, CD8-BUV563, CD45RO-BV650, and CD69-BUV737 at optimally titrated concentrations for 30 minutes at room temperature (Supplemental Table 12). Cells were permeabilized with Cytofix/Cytoperm solution (BD Biosciences) and incubated with Perm Wash buffer containing CD40L-FITC and CD137-APC-Cy7 antibodies for 30 minutes at room temperature. Cells were fixed in 1% paraformaldehyde and acquired on the Aurora cytometer (Cytek Biosciences). Compensation and gating were performed using OMIQ software, and frequencies were normalized across batches (each batch containing PBMCs from 4 participants distributed across dose, time point, and stimulation conditions) using the ComBat function in R, including participant group, vaccine dose, and time point as covariates. For each batch, we included a gating control, which contained only CD45-BV570, CD3-BUV395, CD4-BUV805, CD8-BUV563, and CD45RO-BV650 without AIM antibodies. To account for nonspecific background, responses to media were subtracted from the corresponding antigen-stimulated values for each participant, matched by vaccine dose and time point, prior to batch normalization. Vaccine-induced AIM responses were defined as the absolute change (Δ) between 14 and 0 dpv (Δ14–0 dpv). See Supplemental Table 12 for information on all antibodies used.

### Statistics.

A minimal sample size criterion of *n* greater than 10/p (where p is the number of predictors) was used to ensure sufficient power for regression analysis of vaccine dose. Analysis of titer and HA antibody data was performed using 2-sided, nonparametric tests, as titer data were observed to follow a non-normal distribution in agreement with prior reports (53, 54). For comparisons between time point (e.g., 0 vs. 28 dpv) paired, Wilcoxon signed-rank testing was used. In the case of multiple comparisons, *P* values were adjusted using the Benjamini-Hochberg procedure for controlling the FDR. Correlation of cellular and serologic vaccine response data was performed using Spearman’s correlation testing. For categorical data (e.g., sex, race, and frequency of seroprotective titer levels), χ^2^ or Fisher’s exact testing were performed. The study employed a multivariate analysis approach to evaluate and control for the effects of age, HIV status, demographic variables, vaccination history, and baseline immunity on vaccine-induced immune responses when comparing groups with heterogenous composition. When comparing participants’ paired high-dose versus standard-dose vaccine responses at 28 dpv, regression models controlled for differences in the seasons’ baseline (prevaccination) HAI titer and HA-specific IgG levels. Ordinal regression models were used for HAI titers, and ordinary linear regression was applied to HA-specific IgG analyses. Group contrasts, including differences between age groups and HIV status, were assessed using marginal means estimation derived from the regression models. Statistical analyses and graphing were performed in *R* and in GraphPad Prism. Adjusted *P* values less than or equal to 0.05 were considered statistically significant.

### Study approval.

The study was approved by the IRB at the University of Miami (IRB 20200752). Written informed consent was provided by all participants prior to their enrollment in the study.

### Data availability.

Numerical data underlying figures are reported in the Supporting Data Values file. Analytic code and underlying data required to reproduce the multivariate analyses are publicly available (github.com/TH20255/High-Dose-vs-Standard-Dose-Influenza-Vaccine-Analysis-in-HIV.git) (commit ID: f52610e).

## Author contributions

SP and SGP conceived and designed the experiments. PS performed the experiments. JK, SD, PS, SP, RP, TL, and DADP analyzed the data. JK and SD wrote the manuscript. AR coordinated the clinical study and contributed to participant recruitment. SDB contributed to data interpretation and critically revised the manuscript. All authors revised the manuscript and approved it for publication. JK, SD, and TL accessed and verified the data. JK is designated the first co–first author as he took primary responsibility for drafting and revising the manuscript, integrating feedback from all coauthors.

## Conflict of interest

The authors have declared that no conflict of interest exists.

## Funding support

This work is the result of NIH funding, in whole or in part, and is subject to the NIH Public Access Policy. Through acceptance of this federal funding, the NIH has been given a right to make the work publicly available in PubMed Central.

- NIH grant (5R01AG068110) to SGP and SP.
- Miami Center for AIDS Research (CFAR) Laboratory Sciences Core (supported by NIH grant P30AI073961).
- T32 Predoctoral Training in Translational Immunology Program, Department of Microbiology and Immunology at the University of Miami (T32AI162624), provided support to SD.

## Supplementary Material

Supplemental data

ICMJE disclosure forms

Supporting data values

## Figures and Tables

**Table 1:**
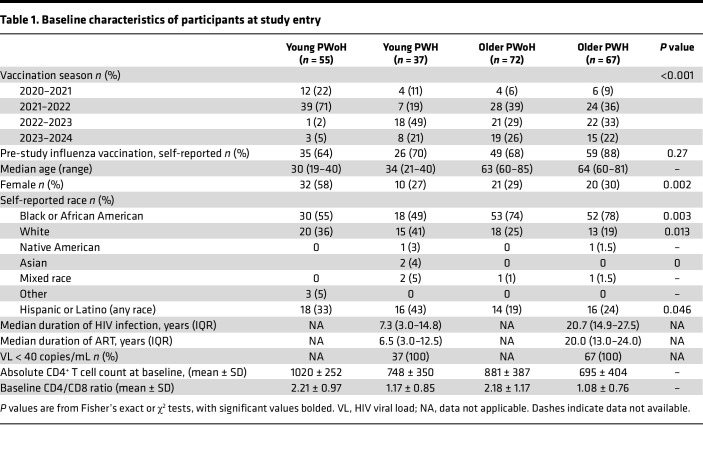
Baseline characteristics of participants at study entry

**Table 2:**
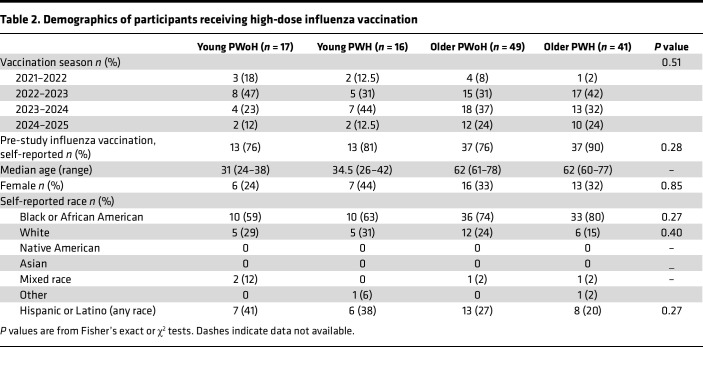
Demographics of participants receiving high-dose influenza vaccination

**Table 3:**
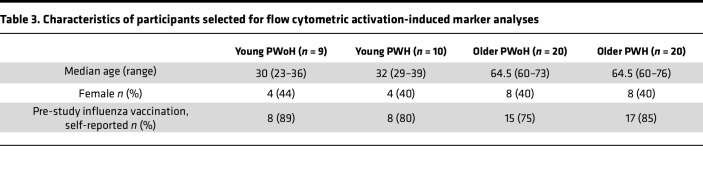
Characteristics of participants selected for flow cytometric activation-induced marker analyses

**Table 4:**
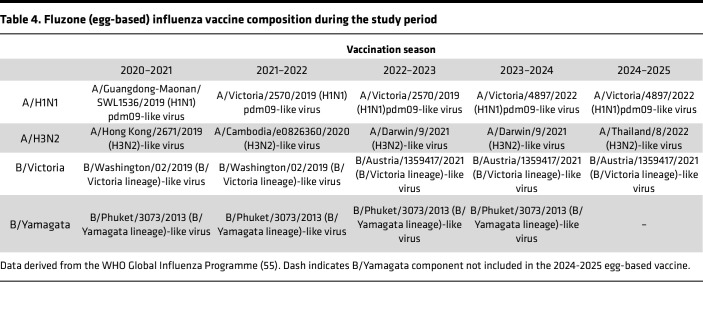
Fluzone (egg-based) influenza vaccine composition during the study period

## Version 1 03/05/2026

In-Press Preview

## Version 2 04/22/2026

Electronic publication
