# Supplementary material for: High-dose influenza vaccine augments serological and cellular immunity of older people with HIV
Source: JCI Insight. 2026 Mar 5;11(8):e199232. doi: 10.1172/jci.insight.199232 (PMC13135403; doi:10.1172/jci.insight.199232)
Supplement: Supplemental data [file jciinsight-11-199232-s210.pdf]

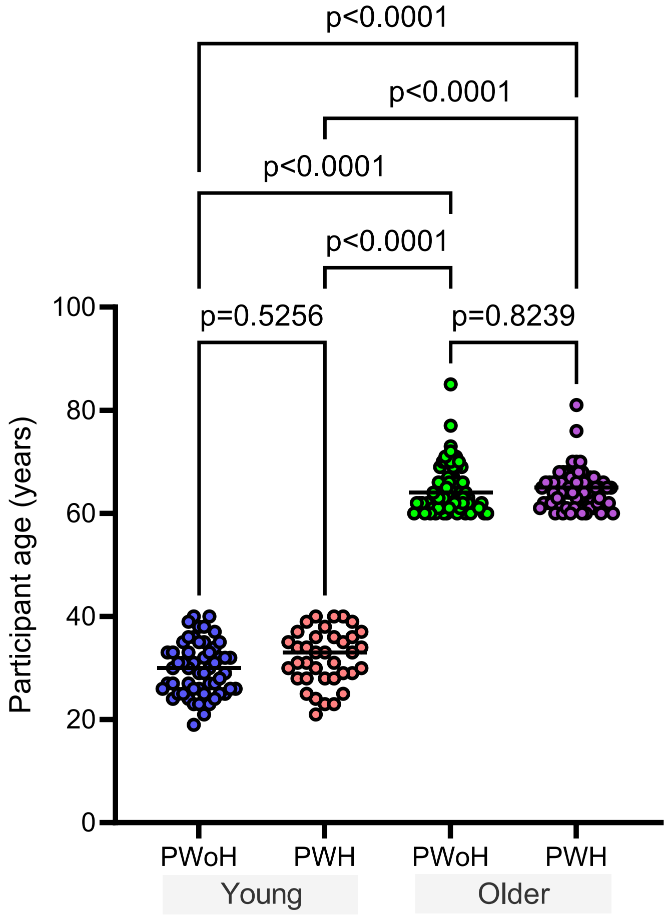

Supplementary Figure 1: Age distribution of study participants. Dot plot showing age (years) for young PWoH (n=55, blue), young PWH (n=37, red), older PWoH (n=72, green), and older PWH (n=67, purple). Horizontal lines indicate group medians. P values are from a Kruskal-Wallis test with FDR-adjusted post-hoc multiple comparisons.

.

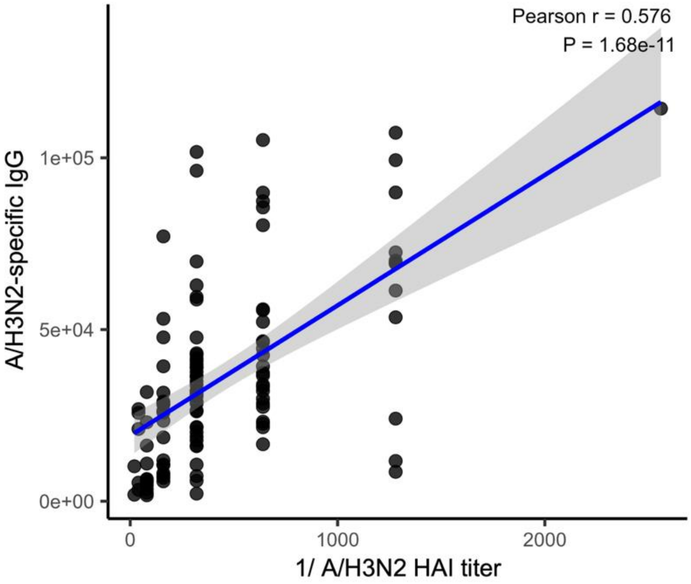

Supplementary Figure 2: A/H3N2 HA-specific IgG is a correlate of A/H3N2 HAI titer at 28 days post-standard-dose vaccination. Pearson correlation between A/H3N2-specific IgG and HAI titer at 28 days post-standard-dose vaccination for a subset of 115 participants vaccinated during the 2020-2021 and 2021-2022 seasons: young PWoH, n=45; young PWH, n=11; older PWoH, n=30; and older PWH, n=29. A linear regression line and its 95% confidence interval are displayed.

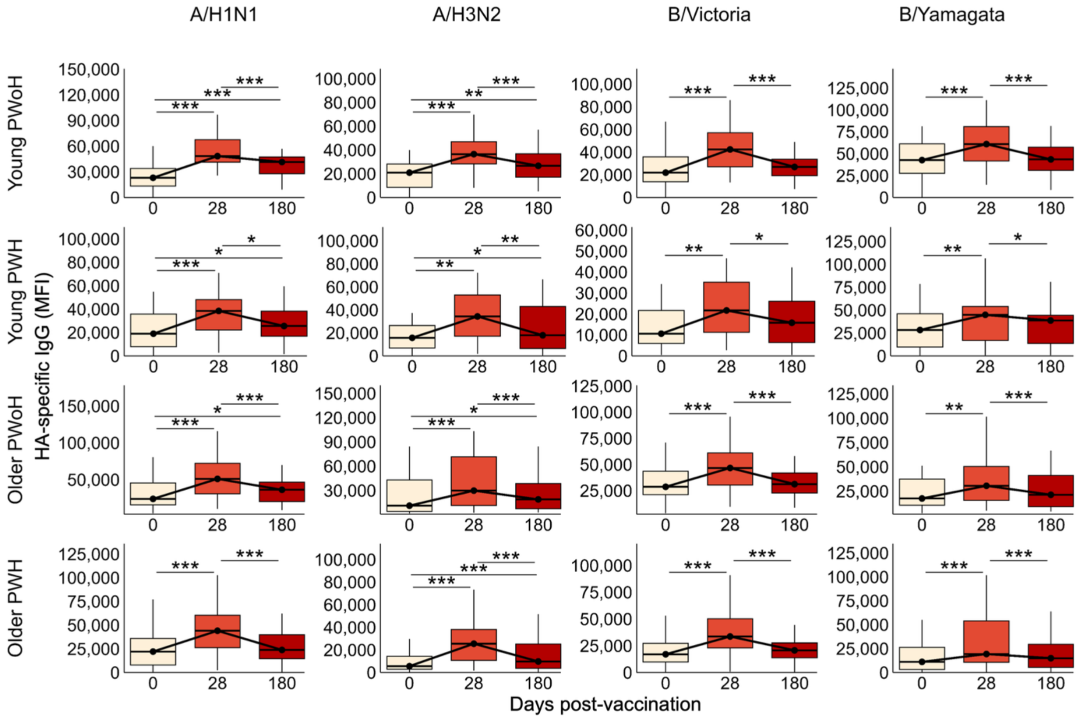

Supplementary Figure 3: HA-specific IgG responses at 0-, 28-, and 180- days post-standard-dose vaccination. Influenza HA-specific IgG levels (mean fluorescence intensity, MFI) of young PWoH (n=30), young PWH (n=20), older PWoH (n=35), and older PWH (n=49) at 0, 28, and 180 days after standard-dose influenza vaccination. P values are from Wilcoxon signed-rank tests between the three timepoints, adjusted for multiple comparisons by the Benjamini-Hochberg method. * = p<0.05; ** = p<0.01; *** = p<0.001; unlabeled comparisons are not significant.

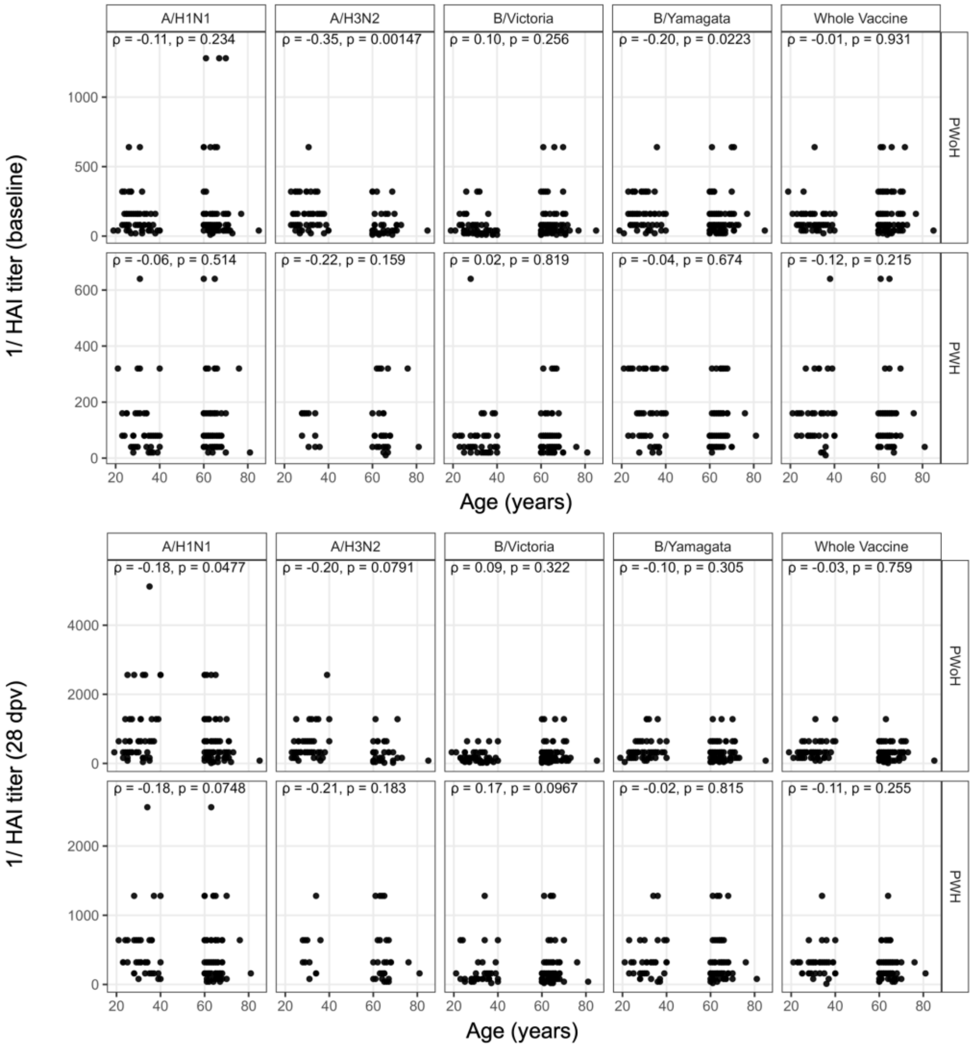

Supplementary Figure 4: Correlation between participant age and HAI titer. Plots show Spearman correlations for HAI titer and participant age at 0- (top) and 28- (bottom) days post-vaccination (dpv), separated by HIV status (PWoH, n=118-127; PWH, n=100-106). A/H3N2 HAI titers were available for a subset of participants vaccinated during the 2020-2021 and 2021-2022 seasons (PWoH, n=76-81; PWH, n=40-41).

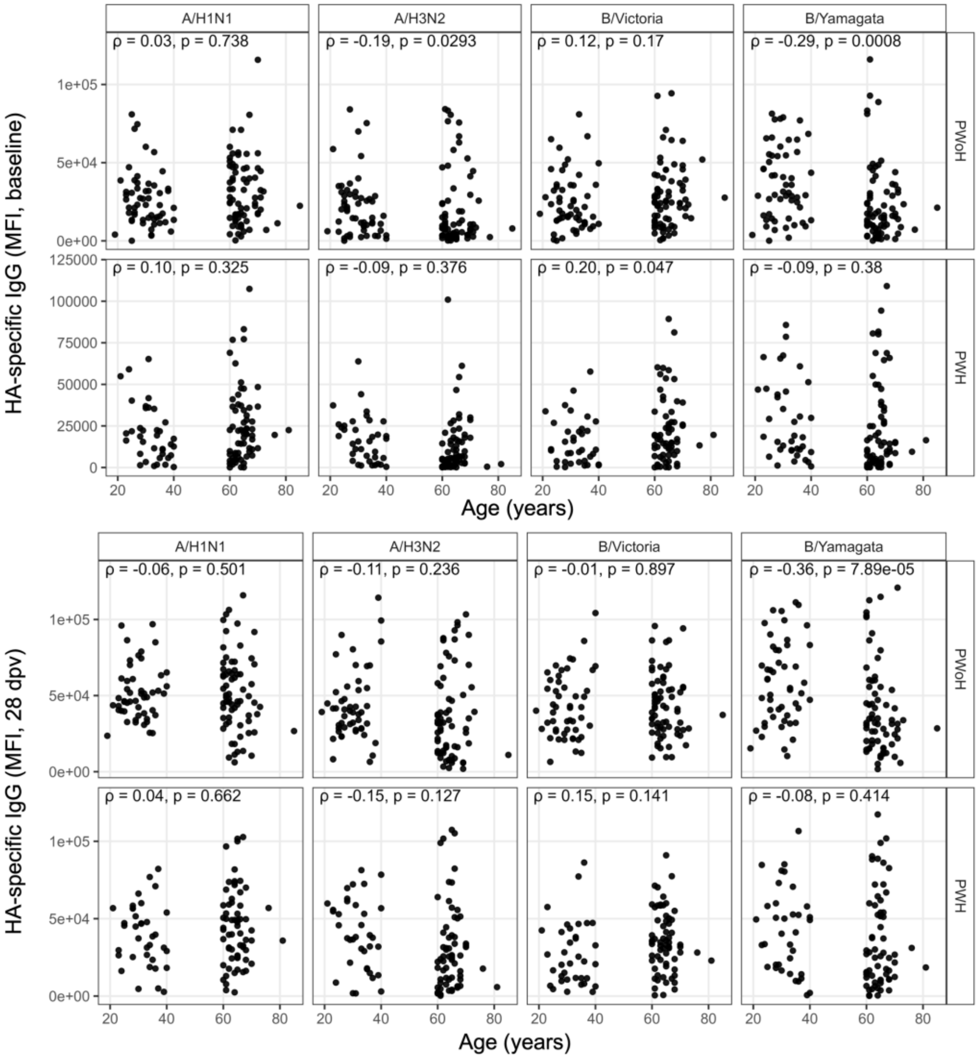

Supplementary Figure 5: Correlation between participant age and HA-specific IgG levels. Plots show Spearman correlations for HA-specific IgG levels (mean fluorescence intensity, MFI) and participant age at 0- (top) and 28- (bottom) days post-vaccination (dpv), separated by HIV status (PWoH, n=118-127; PWH, n=101-104).

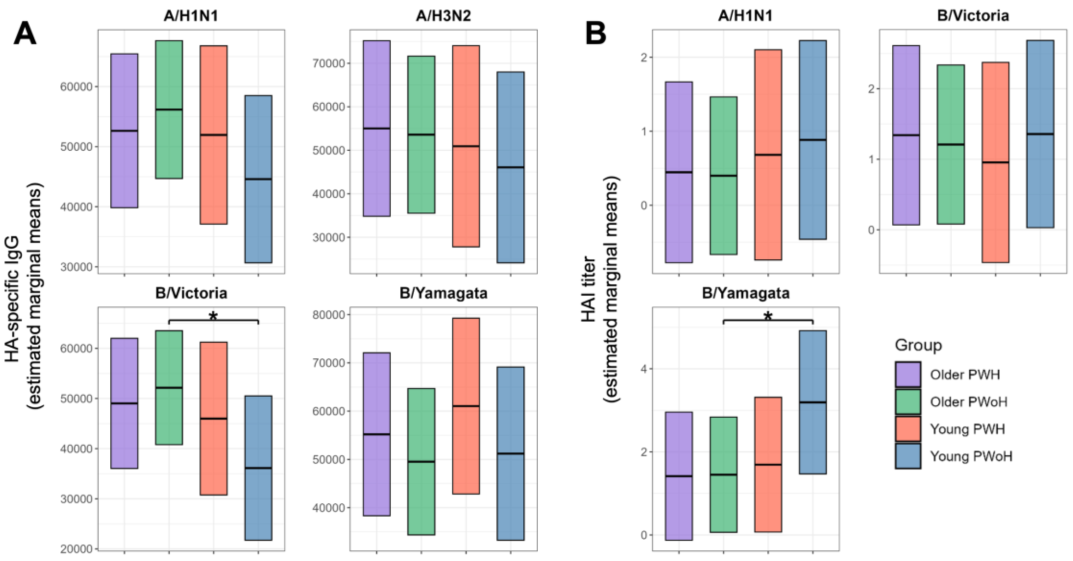

Supplementary Figure 6: Multivariate comparison of group vaccine responses at 28- days post-high-dose vaccination. (**A**) HA-specific IgG levels and (**B**) influenza HAI titer at 28 days post-high-dose vaccination of young PWoH (n=17), young PWH (n=14-15), older PWoH (n=45-46), and older PWH (n=37-39). B/Yamagata HAI titers were not assessed in participants administered trivalent vaccine during the 2024-2025 influenza season (young PWoH, n=2; young PWH, n=2; older PWoH, n=12; older PWH, n=10). Plots display estimated marginal means from a linear regression model controlling for the effects of baseline vaccine responses, previous influenza vaccine history, and demographic variables. * = $p<0.05$.

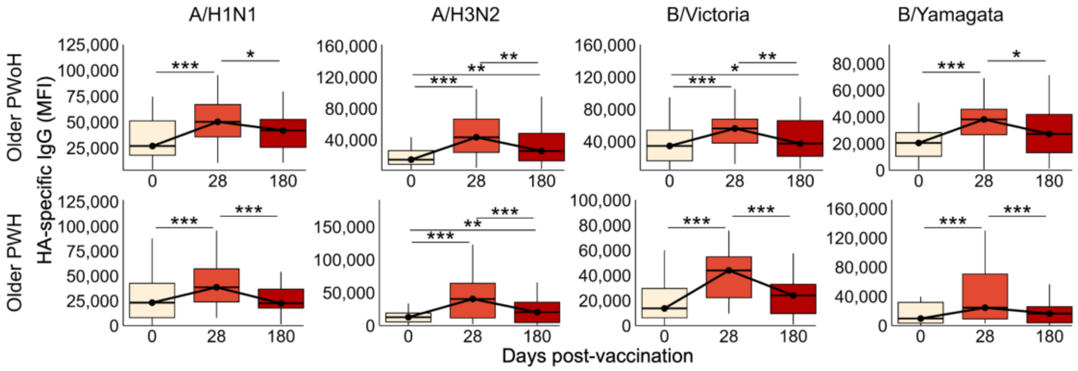

Supplementary Figure 7: HA-specific IgG responses at 0-, 28-, and 180- days post-high-dose vaccination. Influenza HA-specific IgG levels (mean fluorescence intensity, MFI) of older PWoH (n=26), and older PWH (n=25) at 0, 28, and 180 days after high-dose influenza vaccination. B/Yamagata responses were not assessed in participants administered trivalent vaccine during the 2024-2025 influenza season (older PWoH, n=12; older PWH, n=10). P values are from Wilcoxon signed-rank tests between the three timepoints, adjusted for multiple comparisons by the Benjamini-Hochberg method. * = p<0.05; ** = p<0.01; *** = p<0.001; unlabeled comparisons are not significant.

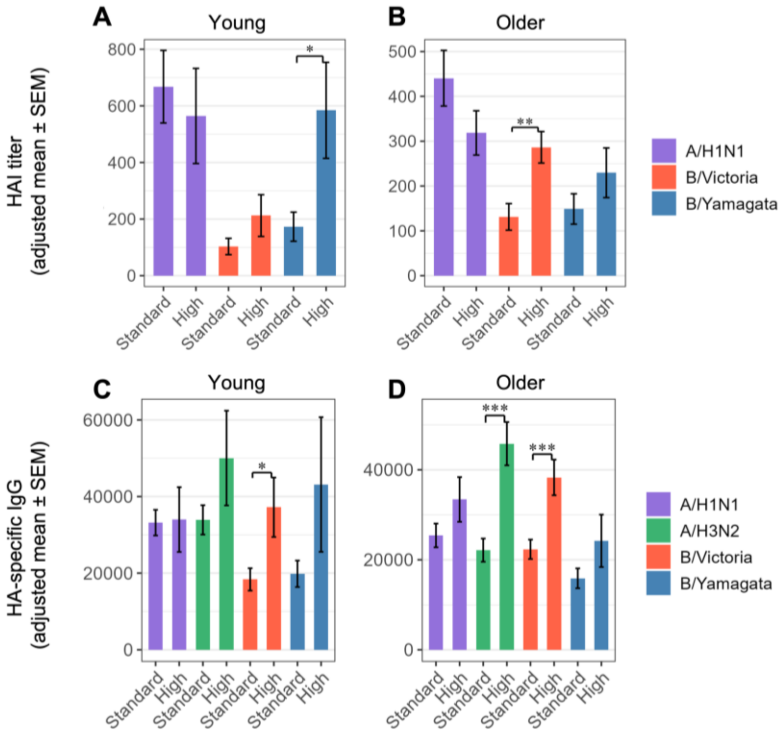

Supplementary Figure 8: Comparison of high-dose vs. standard-dose influenza vaccine responses of young and older study participants. Bar graphs depict estimated marginal means (EMM) and standard errors of high-dose (“High”) vs. standard-dose (“Standard”) influenza vaccine responses at 28 days post-vaccination. EMMs are from a mixed-effects model controlling for baseline (pre-vaccination) immune responses. (**A**-**B**) HAI titer against A/H1N1, B/Victoria (older, n=70; young, n=25), and B/Yamagata (older, n=58; young, n=24). (**C**-**D**) IgG specific to A/H1N1, A/H3N2, B/Victoria (older, n=72; young, n=27), and B/Yamagata (older, n=60; young, n=26). * = p<0.05; ** = p<0.01; *** = p<0.001.

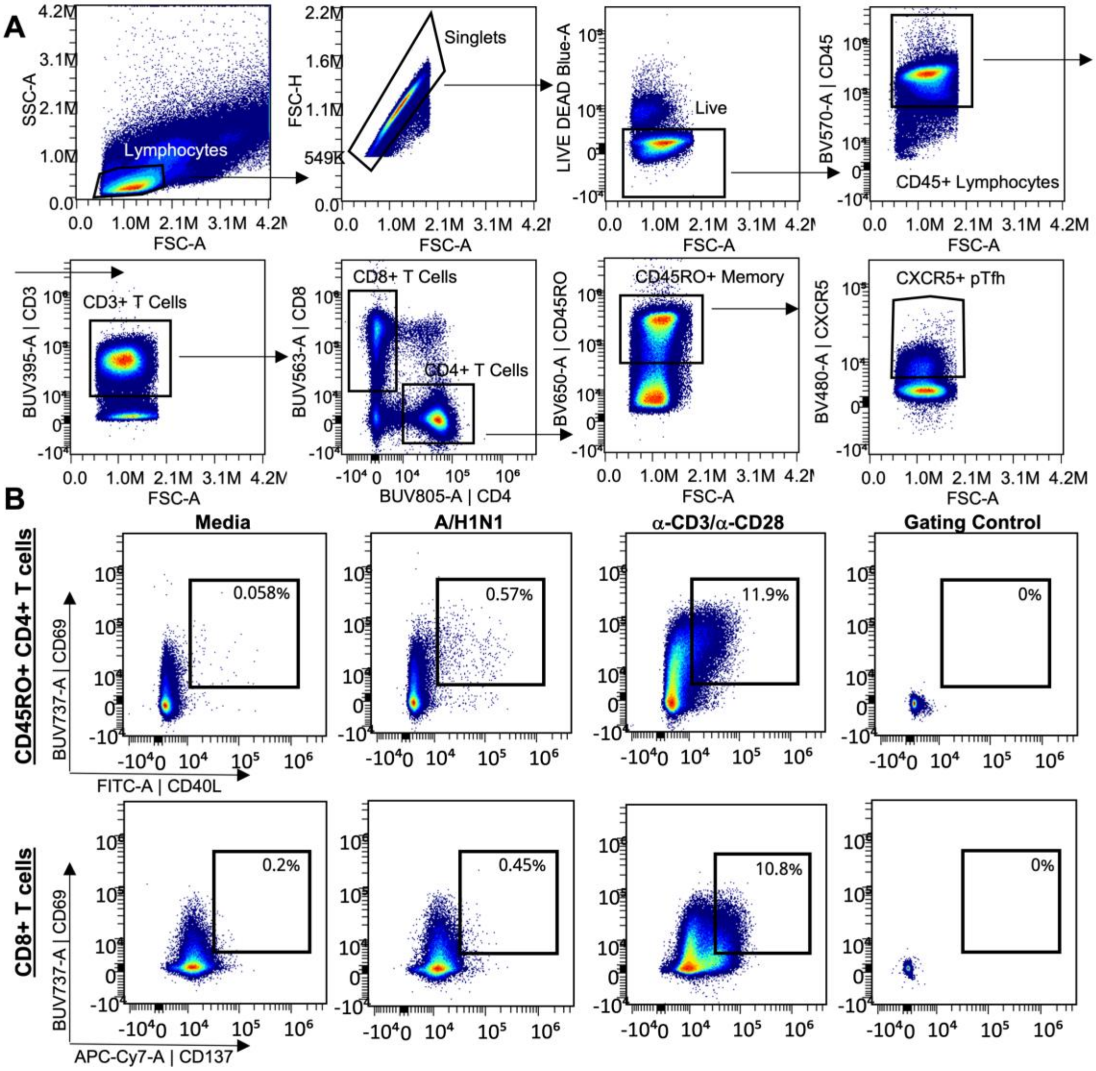

Supplementary Figure 9: Representative flow cytometry plots. (**A**) Gating strategy for CD8+, CD45RO+ memory CD4+, and CD45RO+ CXCR5+ peripheral T follicular helper (pTfh) cells. (**B**) Representative antigen-induced marker (AIM) responses in CD8+ and memory CD4+ T cells following 12-hour stimulation with media, A/H1N1 antigen, or α-CD3/α-CD28. The gating control was obtained from α-CD3/α-CD28-stimulated PBMCs stained with lineage markers in the absence of AIM antibodies.

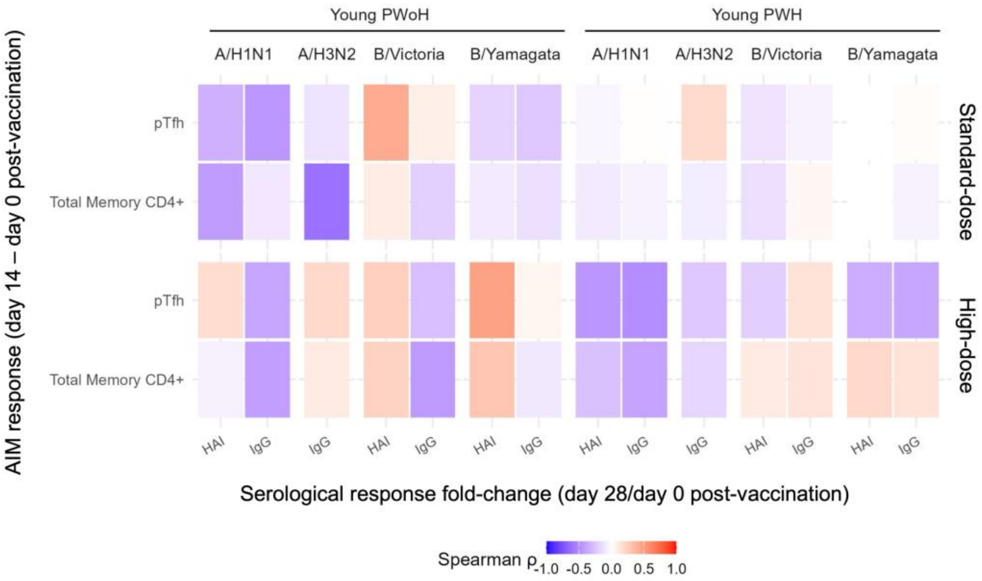

Supplementary Figure 10: Correlation of CD4+ T cell and pTfh AIM responses with influenza seroprotection among young participants. Heatmap showing Spearman correlations between antigen-induced activation marker (AIM) responses in peripheral T follicular helper (pTfh) cells and total memory CD4+ T cells, and serological vaccine responses in young PWoH (n=9) and young PWH (n=10). AIM responses were calculated as media-adjusted, batched normalized frequencies of CD69+CD40L+ cells at day 14 post-vaccination relative to baseline (day 0). Serological responses were quantified as hemagglutination inhibition (HAI) titer or HA-specific IgG fold-change from day 0 to day 28, following standard- or high-dose influenza vaccination. * = $p<0.05$; unlabeled comparisons are not significant.

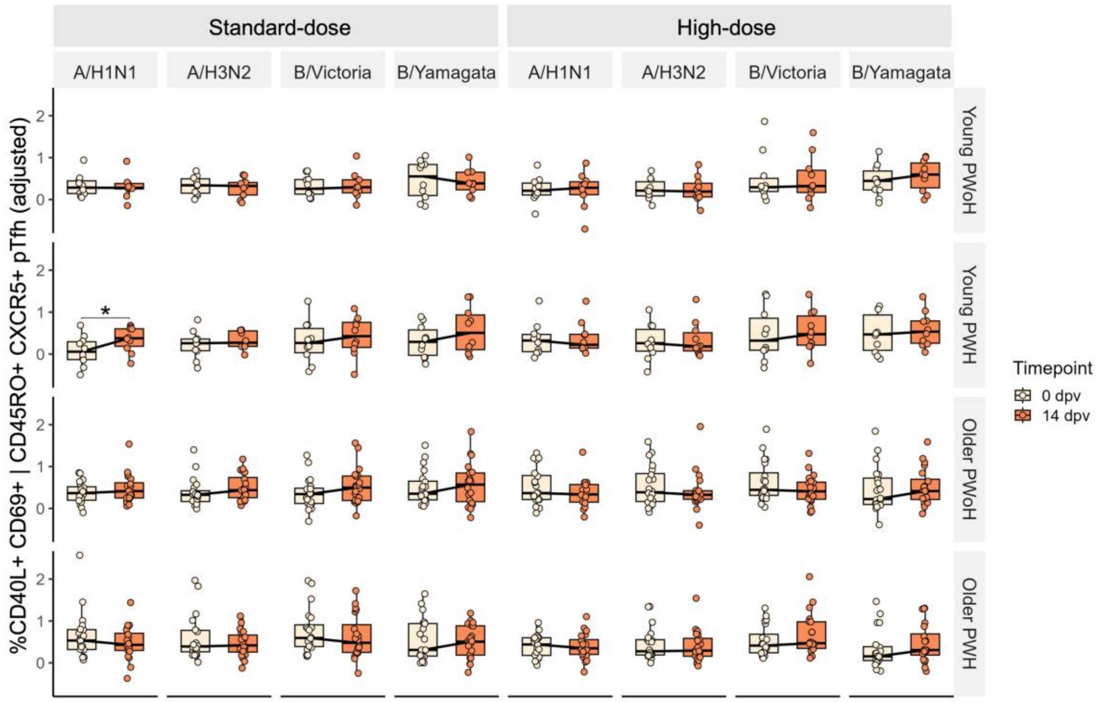

Supplementary Figure 11: Peripheral T follicular helper cell antigen-induced marker responses following standard- and high-dose influenza vaccination. Media-adjusted, batch-normalized frequencies of CD40L+CD69+ peripheral T follicular helper (pTfh) cells are shown at 0- and 14-days post-vaccination (dpv). AIM responses were assessed in young PWoH (n=9), young PWH (n=10), older PWoH (n=20), and older PWH (n=20) following 12-hour stimulation of PBMCs with media or influenza antigens matched to the participants' seasonal influenza vaccine strain. P values are from Wilcoxon signed-rank tests; * = $p<0.05$.

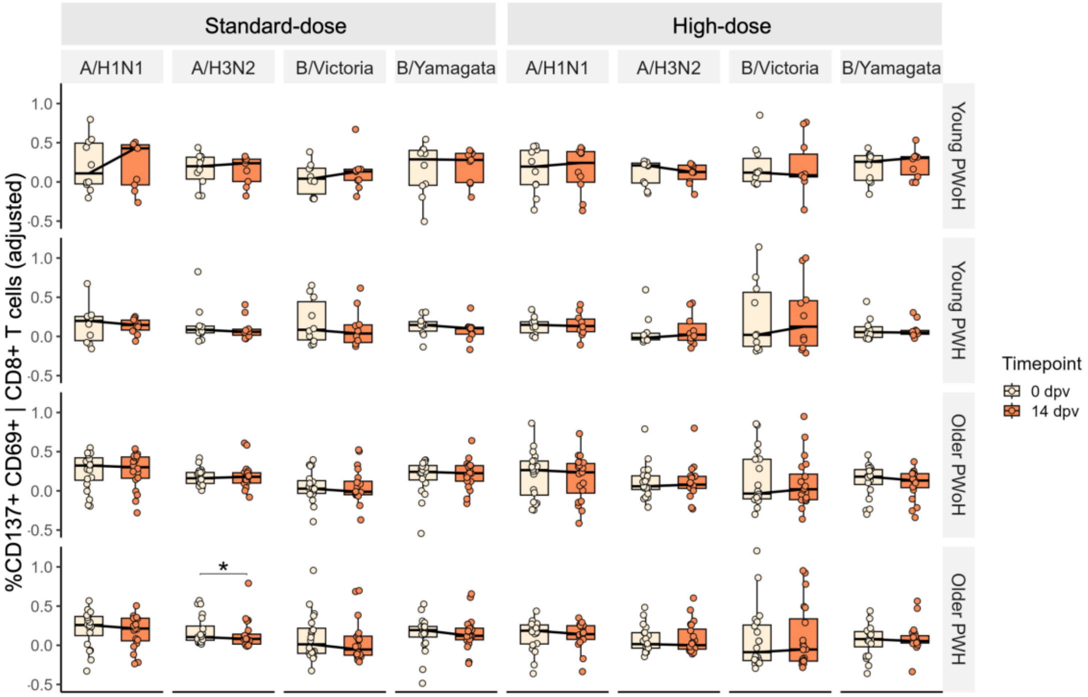

Supplementary Figure 12: CD8+ T cell antigen-induced marker responses following standard- and high-dose influenza vaccination. Media-adjusted, batch-normalized frequencies of CD137+ CD69+ CD8+ T cells are shown at 0- and 14-days post-vaccination (dpv). AIM responses were assessed in young PWoH (n=9), young PWH (n=10), older PWoH (n=20), and older PWH (n=20) following 12-hour stimulation of PBMCs with media or influenza antigens matched to the participants' seasonal influenza vaccine strain. P values are from Wilcoxon signed-rank tests; * = p<0.05.

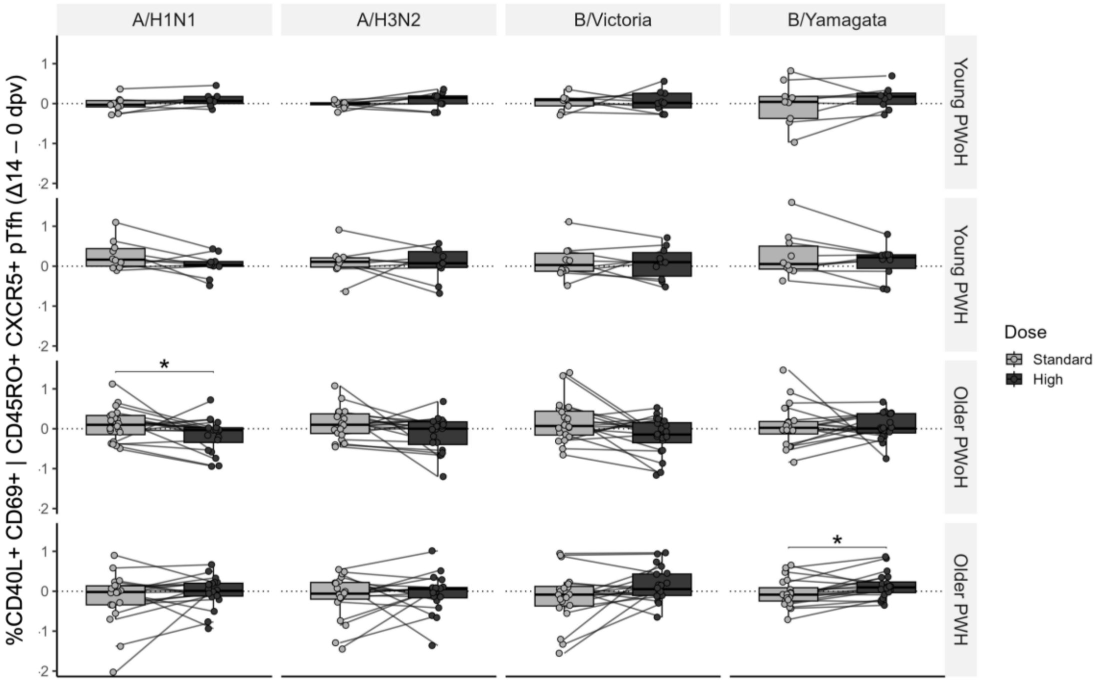

Supplementary Figure 13: Change in peripheral T follicular helper cell antigen-induced marker responses following standard- versus high-dose influenza vaccination. Media-adjusted, batch-normalized frequencies of CD40L+CD69+ peripheral T follicular helper (pTfh) cells were measured at 0- and 14-days post-vaccination (dpv) after standard- and high-dose influenza vaccination, and the change (Δ14 – 0 dpv) is plotted. AIM responses were assessed in young PWoH (n=9), young PWH (n=10), older PWoH (n=20), and older PWH (n=20) following 12-hour stimulation of PBMCs with media or influenza antigens matched to the participants' seasonal influenza vaccine strain. P values are from Wilcoxon signed-rank tests; * = p<0.05.

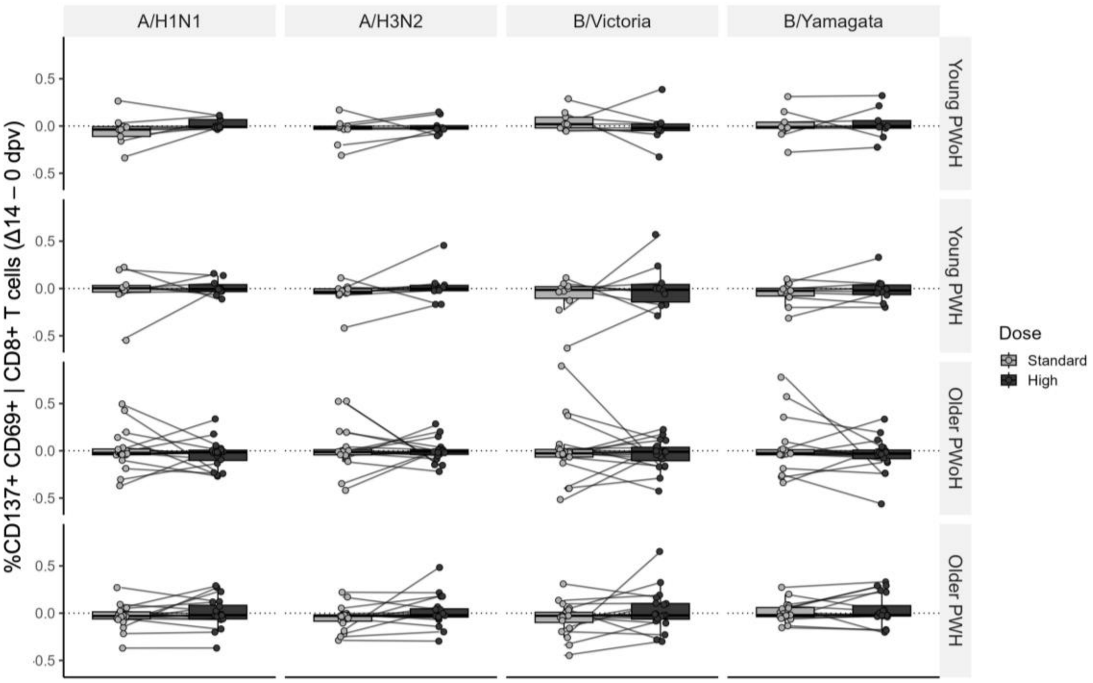

Supplementary Figure 14: Change in CD8+ T cell antigen-induced marker responses following standard- versus high-dose influenza vaccination. Media-adjusted, batch-normalized frequencies of CD137+ CD69+ CD8+ T cells were measured at 0- and 14-days post-vaccination (dpv) after standard- and high-dose influenza vaccination, and the change (Δ14 – 0 dpv) is plotted. AIM responses were assessed in young PWoH (n=9), young PWH (n=10), older PWoH (n=20), and older PWH (n=20) following 12-hour stimulation of PBMCs with media or influenza antigens matched to the participants' seasonal influenza vaccine strain. P values are from Wilcoxon signed-rank tests; * = p<0.05.

Supplementary Table 1: Self-reported comorbidities at study entry.

	**Young PWoH (n=52)**	**Young PWH (n=28)**	**Older PWoH (n=52)**	**Older PWH (n=52)**
Hypertension n (%)	-	1 (4)	14 (27)	15 (29)
Diabetes n (%)	1 (2)	1 (4)	11 (21)	13 (25)
Hyperlipidemia n (%)	-	1 (4)	16 (31)	12 (23)
History of heart attack or stroke n (%)	2 (4)	1 (4)	3 (6)	3 (6)

Self-reported comorbidity data were available for n=184/231 study participants.

Supplementary Table 2: Frequency of HAI titers at ≥ 1:40 and ≥ 1:80 among participants at the time of study enrollment (0 days post-vaccination).

			A/H1N1		
	**Young PWoH**	**Young PWH**	**Older PWoH**	**Older PWH**	**P**
**Total n**	52	37	72	66	
**n ≥ 40 (%)**	48 (92.3)	33 (89.2)	68 (94.4)	62 (93.9)	0.76
**n ≥ 80 (%)**	36 (69.2)	27 (73.0)	52 (72.2)	45 (68.2)	0.93
			**A/H3N2**		
	**Young PWoH**	**Young PWH**	**Older PWoH**	**Older PWH**	**P**
**Total n**	48	11	32	29	
**n ≥ 40 (%)**	45 (93.8)	11 (100)	24 (75.0)	26 (90.0)	0.06
**n ≥ 80 (%)**	39 (81.3)	8 (72.7)	15 (46.9)	19 (65.5)	**0.01**
			**B/Victoria**		
	**Young PWoH**	**Young PWH**	**Older PWoH**	**Older PWH**	**P**
**Total n**	52	37	72	66	
**n ≥ 40 (%)**	31 (59.6)	27 (73.0)	55 (76.4)	52 (78.8)	0.11
**n ≥ 80 (%)**	19 (36.5)	16 (43.2)	36 (50.0)	33 (50.0)	0.41
			**B/Yamagata**		
	**Young PWoH**	**Young PWH**	**Older PWoH**	**Older PWH**	**P**
**Total n**	52	37	72	66	
**n ≥ 40 (%)**	50 (96.2)	34 (92.0)	64 (88.9)	65 (98.5)	0.09
**n ≥ 80 (%)**	43 (82.7)	30 (81.2)	49 (68.1)	52 (78.8)	0.23

Fisher's exact test p values are displayed.

Supplementary Table 3: Frequency of HAI titers at ≥ 1:40 and ≥ 1:80 among participants at 28 days post-standard-dose vaccination.

			A/H1N1		
	**Young PWoH**	**Young PWH**	**Older PWoH**	**Older PWH**	**P**
**Total n**	49	36	68	64	
**n ≥ 40 (%)**	49 (100)	36 (100)	67 (98.5)	64 (100)	>0.99
**n ≥ 80 (%)**	48 (98.0)	36 (100)	63 (92.6)	60 (93.8)	0.30
			**A/H3N2**		
	**Young PWoH**	**Young PWH**	**Older PWoH**	**Older PWH**	**P**
**Total n**	45	11	30	29	
**n ≥ 40 (%)**	45 (100)	11 (100)	27 (90.0)	29 (100)	0.06
**n ≥ 80 (%)**	45 (100)	11 (100)	26 (86.7)	26 (89.7)	**0.04**
			**B/Victoria**		
	**Young PWoH**	**Young PWH**	**Older PWoH**	**Older PWH**	**P**
**Total n**	49	36	68	64	
**n ≥ 40 (%)**	46 (93.9)	35 (97.2)	66 (97.1)	62 (96.9)	0.83
**n ≥ 80 (%)**	40 (81.6)	30 (83.3)	61 (89.7)	53 (82.8)	0.55
			**B/Yamagata**		
	**Young PWoH**	**Young PWH**	**Older PWoH**	**Older PWH**	**P**
**Total n**	49	36	68	64	
**n ≥ 40 (%)**	49 (100)	36 (100)	68 (100)	63 (98.4)	0.69
**n ≥ 80 (%)**	48 (98.0)	34 (94.4)	63 (92.6)	61 (95.3)	0.68

Fisher's exact test p values are displayed.

Supplementary Table 4: Frequency of HAI titers at ≥ 1:40 and ≥ 1:80 among participants at 180 days post-standard-dose vaccination.

			A/H1N1		
	**Young PWoH**	**Young PWH**	**Older PWoH**	**Older PWH**	**P**
**Total n**	21	19	25	45	
**n ≥ 40 (%)**	21 (100)	19 (100)	23 (92.0)	41 (91.1)	0.40
**n ≥ 80 (%)**	21 (100)	19 (100)	22 (88.0)	38 (84.4)	0.08
			**A/H3N2**		
	**Young PWoH**	**Young PWH**	**Older PWoH**	**Older PWH**	**P**
**Total n**	-	-	-	-	
**n ≥ 40 (%)**	-	-	-	-	-
**n ≥ 80 (%)**	-	-	-	-	-
			**B/Victoria**		
	**Young PWoH**	**Young PWH**	**Older PWoH**	**Older PWH**	**P**
**Total n**	21	19	25	45	
**n ≥ 40 (%)**	20 (95.2)	12 (63.2)	24 (96.0)	44 (97.8)	**0.004**
**n ≥ 80 (%)**	15 (71.4)	8 (42.1)	18 (72.0)	35 (77.8)	0.052
			**B/Yamagata**		
	**Young PWoH**	**Young PWH**	**Older PWoH**	**Older PWH**	**P**
**Total n**	21	19	24	45	
**n ≥ 40 (%)**	21 (100)	19 (100)	23 (95.8)	44 (97.8)	>0.99
**n ≥ 80 (%)**	21 (100)	15 (78.9)	17 (70.8)	41 (91.1)	**0.01**

Fisher's exact test p values are displayed.

Supplementary Table 5: Regression of demographic characteristics and prior influenza vaccination on day 28 post-standard-dose vaccination HAI titers and HA-specific IgG responses.

		HAI Titer		HA-Specific IgG (MFI)	
**Predictor**	**Antigen**	**OR (95%CI)**	**P Value**	**β (95%CI)**	**P Value**
**Sex (male)**	A/H1N1	0.95 (0.56, 1.60)	0.80	1577 (-3549, 6703)	0.50
	A/H3N2	-	-	4478 (-2224, 11,179)	0.20
	B/Victoria	1.0 (0.58, 1.70)	>0.90	2955 (-1544, 7455)	0.20
	B/Yamagata	0.82 (0.48, 1.41)	0.50	3602 (-1599, 8804)	0.20
**Race (Black/African American)**	A/H1N1	1.76 (0.55, 5.54)	0.40	7838 (-3961, 19,637)	0.050
	A/H3N2	-	-	-8345 (-23,785, 7095)	**0.006**
	B/Victoria	0.53 (0.16, 1.71)	0.70	-7506 (-17,797, 2785)	**0.015**
	B/Yamagata	0.83 (0.25, 2.80)	0.60	-1532 (-13,532, 10,468)	0.20
**Race (White)**	A/H1N1	2.27 (0.75, 6.92)	0.40	11,643 (309, 22,977)	0.050
	A/H3N2	-	-	8282 (-6557, 23,121)	**0.006**
	B/Victoria	0.52 (0.16, 1.62)	0.70	2261 (-7629, 12,150)	**0.015**
	B/Yamagata	0.65 (0.20, 2.05)	0.60	6164 (-5355, 17,683)	0.20
**Ethnicity Non-Hispanic/Non-Latino**	A/H1N1	1.19 (0.54, 2.61)	0.70	1996 (-5543, 9535)	0.60
	A/H3N2	-	-	11,741 (1827, 21,654)	**0.021**
	B/Victoria	0.84 (0.38, 1.86)	0.70	5043 (-1555, 11,641)	0.13
	B/Yamagata	0.73 (0.33, 1.63)	0.40	3789 (-3833, 11,460)	0.30
**Previous flu vaccination=Yes**	A/H1N1	0.54 (0.16, 1.80)	0.50	-8512 (-14,405, -2618)	**0.005**
	A/H3N2	-	-	-6304 (-14,005, 1396)	0.20
	B/Victoria	1.03 (0.56, 1.88)	0.20	-9357 (-14,545, -4170)	**<0.001**
	B/Yamagata	0.36 (0.19, 0.68)	**0.002**	-11,234 (-17,215, -5253)	**<0.001**

Supplementary Table 6: Regression of age group and HIV status on day 28 post-standard-dose vaccination HAI titers and HA-specific IgG responses.

		HAI Titer		HA-Specific IgG (MFI)	
**Predictor**	**Antigen**	**OR (95%CI)**	**P Value**	**β (95%CI)**	**P Value**
**Age group (young)**	A/H1N1	1.90 (1.13, 3.23)	**0.016**	1587 (-3539, 6712)	0.5
	A/H3N2	-	-	4701 (-1936, 11,339)	0.2
	B/Victoria	0.71 (0.42, 1.21)	0.2	-2127 (-6582, 2328)	0.3
	B/Yamagata	0.93 (0.53, 1.61)	0.8	1903 (-3355, 7160)	0.5
**HIV status (PWH)**	A/H1N1	0.61 (0.37, 1.01)	0.055	-3583 (-8508, 1341)	0.2
	A/H3N2	-	-	-4.2 (-6421, 6413)	>0.9
	B/Victoria	0.99 (0.60, 1.65)	0.9	-4332 (-8686, 23)	0.051
	B/Yamagata	0.92 (0.55, 1.53)	0.8	-4142 (-9097, 812)	0.1

Models were adjusted for sex, race, ethnicity, prior-season influenza vaccination, and baseline (pre-vaccination) antibody responses. Analyses included young (n=85) and older (n=131-132) individuals, consisting of PWoH (n=118) and PWH (n=98-99).

Supplementary Table 7: Frequency of HAI titers at ≥1:40 and ≥1:80 among participants at the high-dose season baseline (0 days post-vaccination)

			A/H1N1		
	**Young PWoH**	**Young PWH**	**Older PWoH**	**Older PWH**	**P**
**Total n**	17	16	49	41	
**n ≥ 40 (%)**	17 (100)	15 (93.8)	47 (95.9)	38 (92.7)	0.72
**n ≥ 80 (%)**	15 (88.2)	12 (75.0)	42 (85.7)	29 (70.7)	0.25
			**B/Victoria**		
	**Young PWoH**	**Young PWH**	**Older PWoH**	**Older PWH**	**P**
**Total n**	17	16	49	41	
**n ≥ 40 (%)**	12 (70.6)	12 (75.0)	44 (90.0)	33 (80.5)	0.21
**n ≥ 80 (%)**	6 (35.3)	7 (43.8)	27 (55.1)	19 (46.3)	0.54
			**B/Yamagata**		
	**Young PWoH**	**Young PWH**	**Older PWoH**	**Older PWH**	**P**
**Total n**	15	14	37	31	
**n ≥ 40 (%)**	14 (93.3)	12 (85.7)	32 (86.5)	29 (93.5)	0.71
**n ≥ 80 (%)**	14 (93.3)	11 (78.6)	27 (73.0)	26 (83.9)	0.39

Fisher's exact test p values are displayed.

Supplementary Table 8: Frequency of HAI titers at ≥1:40 and ≥1:80 among participants at 28 days post-high-dose vaccination.

	Young PWoH	Young PWH	Older PWoH	Older PWH	P
		**A/H1N1**			
	**Young PWoH**	**Young PWH**	**Older PWoH**	**Older PWH**	**P**
**Total n**	17	14	47	38	
**n ≥ 40 (%)**	17 (100)	14 (100)	47 (100)	38 (100)	>0.99
**n ≥ 80 (%)**	17 (100)	12 (85.7)	47 (100)	38 (100)	**0.01**
		**B/Victoria**			
	**Young PWoH**	**Young PWH**	**Older PWoH**	**Older PWH**	**P**
**Total n**	17	14	47	38	
**n ≥ 40 (%)**	16 (94.1)	14 (100)	47 (100)	38 (100)	0.27
**n ≥ 80 (%)**	15 (88.2)	14 (100)	44 (93.6)	35 (92.1)	0.73
		**B/Yamagata**			
	**Young PWoH**	**Young PWH**	**Older PWoH**	**Older PWH**	**P**
**Total n**	15	13	35	28	
**n ≥ 40 (%)**	15 (100)	13 (100)	35 (100)	27 (96.4)	0.62
**n ≥ 80 (%)**	14 (93.3)	13 (100)	33 (94.3)	26 (92.9)	>0.99

Fisher's exact test p values are displayed.

Supplementary Table 9: Regression of demographic characteristics and prior influenza vaccination on day 28 post-high-dose vaccination HAI titers and HA-specific IgG responses.

		HAI Titer		HA-Specific IgG (MFI)	
**Predictor**	**Antigen**	**OR (95%CI)**	**P Value**	**β (95%CI)**	**P Value**
**Sex (male)**	A/H1N1	0.56 (0.26, 1.17)	0.12	1,877 (-5869, 9622)	0.60
	A/H3N2	-	-	9793 (-2299, 21884)	0.11
	B/Victoria	0.65 (0.30, 1.40)	0.30	3288 (-4439, 10,895)	0.40
	B/Yamagata	0.66 (0.27, 1.56)	0.30	6212 (-4003, 16,428)	0.20
**Race (Black/African American)**	A/H1N1	0.40 (0.05, 3.06)	0.60	-9158 (-30,143, 11,826)	0.50
	A/H3N2	-	-	-26,643 (-59,439, 6153)	0.087
	B/Victoria	0.10 (0.01, 0.83)	**0.001**	-9796 (-30,379, 10,787)	0.60
	B/Yamagata	1.10 (0.07, 18.0)	0.14	-9018 (-39,991, 21,956)	0.30
**Race (White)**	A/H1N1	0.72 (0.13, 4.03)	0.60	-2357 (-20,096, 15,381)	0.50
	A/H3N2	-	-	-4591 (-32,517, 23,335)	0.087
	B/Victoria	2.0 (0.43, 9.45)	**0.001**	-4715 (-22,271, 12,841)	0.60
	B/Yamagata	3.94 (0.31, 55.2)	0.14	3608 (-24,751, 31,969)	0.30
**Ethnicity Non-Hispanic/Non-Latino**	A/H1N1	1.45 (0.38, 5.34)	0.60	2218 (-12,066, 16,502)	0.80
	A/H3N2	-	-	15,742 (-5870, 37,354)	0.20
	B/Victoria	11.5 (2.16, 63.4)	**0.004**	3562 (-10,178, 17,303)	0.60
	B/Yamagata	7.79 (1.69, 37.1)	**0.009**	12,668 (-4412, 29,747)	0.14
**Previous flu vaccination=Yes**	A/H1N1	0.39 (0.14, 1.06)	0.20	74 (-9501, 9650)	0.80
	A/H3N2	-	-	-3433 (-18,655, 11,789)	0.40
	B/Victoria	0.42 (0.16, 1.08)	0.20	-1322 (-11,274, 8601)	0.80
	B/Yamagata	0.98 (0.35, 2.71)	0.30	-8003 (-19,801, 3795)	0.20

Supplementary Table 10: Regression of age group and HIV status on day 28 post-high-dose vaccination HAI titers and HA-specific IgG responses.

		HAI Titer		HA-Specific IgG (MFI)	
**Predictor**	**Antigen**	**OR (95%CI)**	**P Value**	**β (95%CI)**	**P Value**
**Age group (young)**	A/H1N1	1.44 (0.62, 3.38)	0.4	-6504 (-14,7811, 1773)	0.12
	A/H3N2	-	-	-5899 (-18,761, 6963)	0.4
	B/Victoria	0.91 (0.41, 1.99)	0.8	-9609 (-18,282, -936)	**0.030**
	B/Yamagata	2.77 (1.07, 7.46)	**0.036**	3678 (-6697, 14,053)	0.5
**HIV status (PWH)**	A/H1N1	0.99 (0.49, 2.00)	>0.9	-549 (-7913, 6815)	0.9
	A/H3N2	-	-	2400 (-9001, 13,801)	0.7
	B/Victoria	0.98 (0.48, 1.98)	>0.9	744 (-6697, 8186)	0.8
	B/Yamagata	0.61 (0.26, 1.41)	0.2	7058 (-2488, 16,604)	0.15

Models were adjusted for sex, race, ethnicity, prior-season influenza vaccination, and baseline (pre-vaccination) antibody responses. Analyses included young (n=31) and older (n=82) individuals, consisting of PWoH (n=62) and PWH (n=51).

Supplementary Table 11: Frequency of HAI titers at ≥1:40 and ≥1:80 among participants at 180 days post-high-dose vaccination.

			A/H1N1		
	**Young PWoH**	**Young PWH**	**Older PWoH**	**Older PWH**	**P**
**Total n**	8	8	25	26	
**n ≥ 40 (%)**	8 (100)	7 (87.5)	25 (100)	24 (92.3)	0.45
**n ≥ 80 (%)**	7 (87.5)	7 (87.5)	21 (84.0)	21 (80.8)	>0.99
			**B/Victoria**		
	**Young PWoH**	**Young PWH**	**Older PWoH**	**Older PWH**	**P**
**Total n**	8	8	25	26	
**n ≥ 40 (%)**	7 (87.5)	6 (75.0)	25 (100)	22 (84.6)	0.06
**n ≥ 80 (%)**	2 (25.0)	4 (50.0)	22 (88.0)	16 (61.5)	**0.004**
			**B/Yamagata**		
	**Young PWoH**	**Young PWH**	**Older PWoH**	**Older PWH**	**P**
**Total n**	8	8	25	26	
**n ≥ 40 (%)**	8 (100)	8 (100)	25 (100)	24 (92.3)	0.71
**n ≥ 80 (%)**	8 (100)	7 (87.5)	22 (88.0)	21 (80.8)	0.74

Fisher’s exact test p values are displayed.

Supplementary Table 12: Information on antibodies used in flow cytometry-based assays.

Target	Clone	Fluorochrome	Vendor	Volume/100 µL test (µL)
CD45	HI30	BV570	BioLegend	5
CD3	SK7	BUV395	BD Biosciences	5
CD4	SK3	BUV805	BD Biosciences	5
CD8	RPA-T8	BUV563	BD Biosciences	0.625
CD45RO	UCHL1	BV650	BD Biosciences	5
CD69	FN50	BUV737	BD Biosciences	0.625
CD40L	2431	FITC	BioLegend	0.625
CD137	41BB	APC-Cy7	BioLegend	1.25
